# Microarray and Pathway Analysis Reveal Distinct Mechanisms Underlying Cannabinoid-Mediated Modulation of LPS-Induced Activation of BV-2 Microglial Cells

**DOI:** 10.1371/journal.pone.0061462

**Published:** 2013-04-24

**Authors:** Ana Juknat, Maciej Pietr, Ewa Kozela, Neta Rimmerman, Rivka Levy, Fuying Gao, Giovanni Coppola, Daniel Geschwind, Zvi Vogel

**Affiliations:** 1 The Dr. Miriam and Sheldon G. Adelson Center for the Biology of Addictive Diseases, Sackler Faculty of Medicine, Physiology and Pharmacology Department, Tel Aviv University, Tel Aviv, Israel; 2 Neurobiology Department, Weizmann Institute of Science, Rehovot, Israel; 3 Program in Neurogenetics, Department of Neurology, University of California Los Angeles, Los Angeles, California, United States of America; Loyola University Chicago, United States of America

## Abstract

Cannabinoids are known to exert immunosuppressive activities. However, the mechanisms which contribute to these effects are unknown. Using lipopolysaccharide (LPS) to activate BV-2 microglial cells, we examined how Δ^9^-tetrahydrocannabinol (THC), the major psychoactive component of marijuana, and cannabidiol (CBD) the non-psychoactive component, modulate the inflammatory response. Microarray analysis of genome-wide mRNA levels was performed using Illumina platform and the resulting expression patterns analyzed using the Ingenuity Pathway Analysis to identify functional subsets of genes, and the Ingenuity System Database to denote the gene networks regulated by CBD and THC. From the 5338 transcripts that were differentially expressed across treatments, 400 transcripts were found to be upregulated by LPS, 502 by CBD+LPS and 424 by THC+LPS, while 145 were downregulated by LPS, 297 by CBD+LPS and 149 by THC+LPS, by 2-fold or more (p≤0.005). Results clearly link the effects of CBD and THC to inflammatory signaling pathways and identify new cannabinoid targets in the MAPK pathway (*Dusp1, Dusp8, Dusp2*), cell cycle related (*Cdkn2b, Gadd45a*) as well as JAK/STAT regulatory molecules (*Socs3, Cish, Stat1*). The impact of CBD on LPS-stimulated gene expression was greater than that of THC. We attribute this difference to the fact that CBD highly upregulated several genes encoding negative regulators of both NFκB and AP-1 transcriptional activities, such as *Trib3* and *Dusp1* known to be modulated through Nrf2 activation. The CBD-specific expression profile reflected changes associated with oxidative stress and glutathione depletion via Trib3 and expression of ATF4 target genes. Furthermore, the CBD affected genes were shown to be controlled by nuclear factors usually involved in regulation of stress response and inflammation, mainly via Nrf2/Hmox1 axis and the Nrf2/ATF4-Trib3 pathway. These observations indicate that CBD, and less so THC, induce a cellular stress response and that this response underlies their high immunosuppressant activities.

## Introduction

*Cannabis sativa* extracts (marijuana and hashish) have been used for centuries both as therapeutic agents and recreational drugs, primarily due to their ability to regulate neurobehavioral processes including memory, mood and appetite [1], [2]. The spectra of possible therapeutic uses of marijuana and its active constituents, the cannabinoids, range from handling nausea, vomiting and cachexia (in cancer chemotherapy and AIDS patients) to treatment of chronic pain, glaucoma, epileptic seizures, Parkinsonian tremor as well as multiple sclerosis [1], [2]-[6]. Amongst the 60 different cannabinoids identified from *Cannabis* preparations, Δ^9^-tetrahydrocannabinol (THC), the major psychoactive *Cannabis* constituent, and the non-psychoactive cannabidiol (CBD) are the most abundant and important.

Many of the beneficial effects of cannabinoids have been attributed to their potent immunosuppressive and anti-inflammatory properties [7]–[14]. Additionally, cannabinoids are known to possess pro-apoptotic, neuroprotective and anti-tumor properties [15]–[17]. To date, two cannabinoid receptors have been characterized: the CB_1_ and the CB_2_ receptors. The CB_1_ receptor is highly expressed in neural cells and mediates the psychoactive and addictive activities of cannabinoids, while the CB_2_ receptor is abundantly present in the periphery including the immune system and is involved in cannabinoid immunomodulation [18]–[20]. THC binds to both of these receptors with similar efficiency [21] and has been reported to have effects on both the nervous and the immune systems [7], [11], [18], [22].

In the last years, attention has been turned to several other phytocannabinoids, most notably to CBD. CBD displays a diversity of actions, including anticonvulsive, sedative, hypnotic, antipsychotic, anti-inflammatory and neuroprotective properties [11]–[14], [23]–[28]. CBD, unlike THC, is not an efficient ligand of either CB_1_ or CB_2_ and therefore, is devoid of the unwanted psychotropic effects (mediated via CB_1_) characteristic of marijuana or THC. Thus, the effects of CBD are probably mediated through other receptors/targets, as described below and elsewhere [23], [29], [30].

Phytocannabinoids were reported to affect various populations of immune cells [7], [11]–[14], [31]. Both THC and CBD have been shown to decrease cytokine production in human immune cell lines [32] and to suppress T cell proliferation and their effector functions [12], [33], [34]. In response to stimulation with the bacterial endotoxin lipopolysaccharide (LPS), both monocytes and microglial cultures treated with either THC or CBD produce lower levels of cytokines such as tumor necrosis factor α (TNFα), interleukin-1α (IL-1α), IL-1β and IL-6 [11], [35]. However, the molecular mechanisms involved in these cannabinoid-mediated effects are not yet fully characterized. Eljaschewitsch *et al*., [36] showed that activation of CB_1_ and/or CB_2_ receptors in the murine microglial BV-2 cell line leads to rapid induction of mitogen-activated protein kinase phosphatase-1 (MKP-1) and that this event switches off MAPK signal transduction which was activated by LPS stimulation. Altered adenosine signaling (through inhibition of its uptake) has been reported as a potential non-cannabinoid receptor mechanism by which CBD, but less so THC, can decrease inflammation [37]. Other studies identified the nuclear receptor peroxisome proliferator-activated receptor γ (PPARγ) as a novel intracellular target, which mediates the cannabinoid-associated immunosuppression in a manner that is independent of the known cannabinoid receptors CB_1_ and CB_2_ [38], [39]. Other targets including the G-protein-coupled receptors GPR55 and GPR18 as well as the transient receptor potential (TRP) channels were also suggested [19], [20], [30], [40].

Microglial cells are the resident macrophage-like cells of the CNS. They are highly ramified cells and their processes are very dynamic under non-pathological conditions, actively scanning their environment. These cells have important roles in brain's innate immunity and neuronal homeostasis as well as in neuroinflammatory pathologies [41]. Microglia can be activated by infection, injury or by endogenously released neurotoxic factors and their activation is associated with the production and secretion of a variety of compounds such as cytokines, reactive oxygen species (ROS), reactive nitrogen species, matrix metalloproteinases and prostaglandins. Although microglial activation is considered a protective mechanism involved in the clearance of pathogen infection and in regulating tissue repair and recovery, excessive or chronic activation can lead to harmful effects [42]. Interestingly, the mechanisms that give rise to either the protective or the damaging microglial phenotypes are not fully elucidated. Enhancing the microglial-mediated innate immunity in the CNS and/or preventing the harmful effects associated with their chronic activation may offer new therapeutic approaches for the treatment of brain injury and neurodegenerative diseases [43].

One of the most potent stimuli for microglia activation is the bacterial endotoxin LPS, that mimics infection by Gram-negative bacteria. LPS activates intracellular signaling pathways in a complex way leading to secretion of cytokines and to overexpression of several markers of the immune response. Previous studies reported that LPS stimulation induces gene expression of *TNFα*, *IL-1β*, *IL-6*, *iNOS* and *COX-2* as well as the production of NO and PGE_2_ in primary and BV-2 microglial cell cultures [11], [44], [45]. We reported that CBD reduces the activity of the NF-κB pathway and upregulates the activation of STAT3 transcription factor in LPS-stimulated BV-2 cells, and that both CBD and THC decrease the activation of the LPS-induced STAT1 transcription factor, a key player in IFNβ-dependent pro-inflammatory processes [11]. Moreover, performing comparative microarray analysis of genome-wide mRNA levels in the BV-2 cells, we reported that CBD, but less so THC, shows a specific gene expression profile associated with oxidative stress and glutathione depletion involving the GCN2/eIF2α/p8/ATF4/Chop-Trib3 pathway [13]. Furthermore, the CBD-stimulated genes were shown to be controlled by nuclear factors known to be involved in regulation of stress response and inflammation, mainly via the (EpRE/ARE)-Nrf2/Atf4 system and the Nrf2/Hmox axis. We reported that CBD, but less so THC, affects the expression of genes involved in zinc homeostasis, suggesting that the regulation of zinc levels could have an important role through which CBD may exert its antioxidant and anti-inflammatory effects [14].

Although the inhibitory functions of cannabinoids on LPS-activated NF-κB and IFN-β/STAT proinflammatory pathways and on the secretion of selected cytokines in BV-2 microglial cells has been studied [11], a genome-wide search for cannabinoid molecular targets in LPS-activated BV-2 cells has not yet been performed. We have, therefore, performed gene array studies and comparative gene profiling analysis of BV-2 cells treated with LPS, CBD+LPS or THC+LPS. This approach allowed us to analyze the changes induced by CBD and THC on gene expression patterns in LPS-treated BV-2 cells and to explore the genome-wide interaction network affected by these treatments. In this regard, a structured network knowledge-based approach to analyze genome-wide transcriptional responses in the context of known functional interactions among proteins, small molecules and phenotypes has been established [46]. We applied this analysis to show the interactions and signaling networks elicited by the cannabinoids in LPS-stimulated BV-2 cells. Our results show that CBD is a potent modulator of microglial activation. Identification of the CBD- and THC-regulated genes and related networks provides a molecular basis for understanding the effects of these cannabinoids on LPS-activated microglia.

## Materials and Methods

### Materials

LPS from *Escherichia coli* (serotype 055:B5) was purchased from Sigma (St. Louis, MO, USA). THC and CBD were obtained from the National Institute on Drug Abuse (NIDA; Baltimore, MD, USA). Stock solutions of cannabinoids were prepared in ethanol and diluted into culture medium before experiments. Final ethanol concentration in the medium did not exceed 0.1%.

### Microglial cell culture

The immortalized murine BV-2 microglial cell line was kindly provided by Prof. E.J. Choi from the Korea University (Seoul, Korea). Cells were grown in Dulbecco's modified Eagle's medium (DMEM; Gibco-BRL, Gaithersburg, MD, USA) containing 4.5 g/L glucose, supplemented with 5% fetal calf serum, penicillin (100 U/ml) and streptomycin (100 µg/ml) (Biological Industries Ltd., Kibbutz Beit Haemek, Israel), under a humidified 5% CO_2_ atmosphere at 37°C. Cells were pretreated for 2 h with either THC or CBD (both at 10 µM) followed by addition of LPS (100 ng/ml) for another 4 h.

### Total RNA extraction

Extraction, quantification and quality of extracted RNA were performed and analyzed as described previously [13].

### Microarray transcript analysis

Comparative microarray analysis was performed using the Illumina MouseRef-8 BeadChip platform, as described previously [13]. Four replicates of each experiment were carried out, resulting in four independent microarrays for each individual treatment and controls (24 total arrays). Gene products that were affected by 2-fold or more were further analyzed as described in the Result section. Differentially expressed genes were classified according to their gene ontology (GO; http://www.geneontology.org/; [47]), using **DAVID** Bioinformatics online tools (**D**atabase for **A**nnotation, **V**isualization and **I**ntegrated **D**iscovery; http://david.abcc.ncifcrf.gov/; [48]). Cellular pathway association was analyzed according to the **K**yoto **E**ncyclopedia of **G**enes and **G**enomes (**KEGG**) database (http://www.genome.jp/kegg/) and pathway maps according to **BioCarta** (http://www.biocarta.com/genes/index.asp).

### Ingenuity pathway analysis

Pathway and global functional analyses were performed using **I**ngenuity **P**athway **A**nalysis 6.0 (**IPA**; Ingenuity® Systems, www.ingenuity.com). A data set containing gene identifiers and corresponding expression values was uploaded into the application, and each gene identifier was mapped using the **I**ngenuity **P**athways **K**nowledge **B**ase (**IPKB**). The IPKB analyses identify the biological functions as well as the pathways from the IPA library that are most significant to the data set. Genes from the data sets associated with biological functions or with a canonical pathway in the IPKB, that met the p-value cutoff of 0.005 were used to build the interactome as described below. Fisher's exact test was used to calculate a *p*-value determining the probability that each biological function and/or canonical pathway assigned to this data set was not due to chance alone.

### Interactome/network analysis of differentially expressed genes

The accession number and the ratio (CBD+LPS *versus* LPS and THC+LPS *versus* LPS) for each gene whose expression was changed by at least 2-fold by one of the treatments (focused genes), were uploaded into the IPA system. This IPA application was used to query databases for interactions between sets of differentially expressed genes and all other genes stored in the knowledge base to generate a set of interactive networks taking into consideration canonical pathways, relevant biological interactions as well as cellular and disease processes. The IPA system computes a score for each network according to the fit of the set of the supplied “focus genes”. The *p* value scores for such a set indicate the likelihood of focused genes to belong to a network *versus* those obtained by chance.

### Microarray data validation by quantitative real time PCR

Many of the gene products that were found to be affected using the microarray gene analysis were validated by quantitative real time reverse transcription polymerase chain reaction (qPCR). Primer sets for qPCR were designed using Primer Quest, an online tool provided by Integrated DNA Technologies (http://eu.idtdna.com/Scitools/Applications/Primerquest/) (**Table S1**). Wherever possible, designs with at least one of the primer sequences located on an intron–exon boundary were chosen, thus avoiding co-amplification of minor contaminating amounts of genomic DNA that could be present in the RNA samples. All primers were analyzed using the nucleotide program BLAST to ensure primer specificity for the gene of interest (http://blast.ncbi.nlm.nih.gov/Blast.cgi). cDNA was generated by using the QuantiTect Reverse Transcription kit containg “genomic DNA wipe out” (to eliminate contamination with genomic DNA), according to the manufacturer's instructions (Qiagen, AG, Basel, Switzerland). qPCR was carried out as detailed by Juknat *et al*., [13] using the Rotor-Gene 3000 instrument (Corbett Research, Sydney, Australia). Expression levels of genes of interest were normalized to the reference gene, *β_2_-microglobulin* (*B2m*), whose expression was found not to be affected by the various treatments, and are expressed as fold change using the calculation method described previously [13]. The qPCR experiments were repeated 3 to 4 times using different mRNA batches from independent experiments and reactions were performed in duplicates for each cDNA sample.

### Statistics

qPCR data were plotted as the mean±SEM of 3-4 independent experiments. Statistical significance was assessed using a one-way or two-way ANOVA, followed by Bonferroni post hoc multiple comparison test as implemented with the Statistics Toolbox Software MATLAB, Version 6.1 (R2007b), MathWorks (http://www.mathworks.com/help/toolbox/stats/rn/brasjn_.html). A *p* value <0.05 was defined as statistically significant.

## Results

### Effect of cannabinoids on LPS-stimulated gene expression in BV-2 microglial cells

BV-2 microglial cells were pretreated for 2 h with either THC or CBD (both at 10 µM) followed by the addition of LPS (100 ng/ml) to the incubation medium for another 4 h. The control treatment with cannabinoids alone lasted for 6 h and with LPS alone for 4 h. The choice of these time points for transcriptional profiling was guided by our previous studies [11], [13] as well as by other reports [44], [45] which investigated the general temporal pattern of microglial activation by LPS. Moreover, according to our previous results, neither THC nor CBD treatments (both at 10 µM) significantly affected the viability of the BV-2 microglial cells during this 6 h period [11].

**Figure 1:**
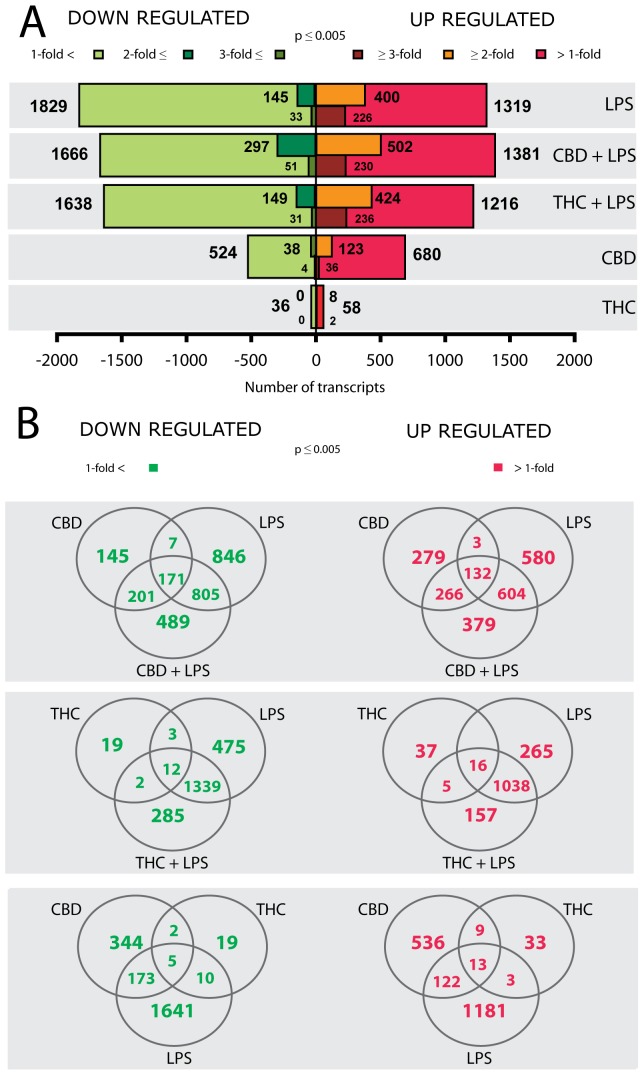
Gene array analysis of the mRNA of BV-2 cells following treatment with LPS and cannabinoids. mRNA was prepared from BV-2 cells treated with LPS and cannabinoids and subjected to array analysis as described in Methods. (A) Number of differentially expressed genes that were either significantly upregulated (red) or downregulated (green) across the different treatment conditions versus control untreated cells (p ≤0.005). The different shades of color represent the indicated fold of change (>1, ≥2 and ≥3). (B) Venn diagrams representing the numbers of BV-2 microglial transcripts that were either upregulated or downregulated at a significance of p≤0.005 following treatments with LPS, CBD and THC, or with a combination of LPS and one of the cannabinoids.

The RNA prepared from these samples (in sextuplicates) was analyzed for changes in transcriptional levels using the MouseRef-8 v1.1 Expression BeadChip Illumina Arrays. Each of these arrays has >24,000 mouse targets based on the NCBI mouse Reference Sequence Database, including 16,287 constitutive exons/islands based on the splice variants in the mouse transcriptome (Molecular Signature Database; MouSDB3) and NCBI LocusLink databases. The results of the analyses of the arrays showed that 32% of the transcripts were consistently “present” in the BV-2 RNA samples across all arrays. Moreover, clustering based on inter-array Pearson correlation coefficient indicated no batch effects. Microarray analysis based on a threshold of p≤ 0.005, revealed that a total of 22% (5338 out of 24,000 transcripts) of the Illumina gene set was differentially regulated across treatments. Of these, 1319 gene probe sets were upregulated and 1829 transcripts were downregulated by the LPS treatment (**Figure 1A**); and from these numbers of genes, 400 transcripts were found to be upregulated and 145 downregulated by LPS by 2-fold or more. When the fold change was set on ≥3-fold (p≤ 0.005), we found that 226 gene products were upregulated and 33 were downregulated by LPS **(** **Figure 1A** **)**. The vast majority of the LPS-affected transcripts (89% or 1181 gene probe sets of the upregulated genes, p≤ 0.005 and 90% or 1641 gene probes of the downregulated transcripts, p≤ 0.005) represented genes that were exclusively responsive to LPS stimulation, and not to treatment with CBD alone or THC alone (**Figure 1B**).

the CBD+LPS treatment was analyzed, 1381 gene probe sets (25.8%) were upregulated and 1666 transcripts (31.2%) were downregulated by CBD+LPS (**Figure 1A**). From these numbers of genes, 379 gene products were found to be upregulated and 489 downregulated exclusively as a response to the combination of CBD+LPS and not affected by LPS or CBD alone (**Figure 1B**). When the fold change was set on ≥2-fold, 502 transcripts were upregulated and 297 gene probe sets were downregulated by the CBD+LPS treatment (**Figure 1A**).

When LPS was applied in the presence of THC, 1216 gene probe sets (22.8%) were upregulated and 1638 transcripts (30.7%) were downregulated; and from these, 424 transcripts were found to be upregulated and 149 downregulated by 2-fold or more (**Figure 1A**). From the 1216 upregulated genes, 157 transcripts were exclusively responsive to THC+LPS treatment alone and from the 1638 downregulated transcripts, 285 gene probes were affected only when THC and LPS were together (**Figure 1B**). When the fold change was set on ≥3-fold, we found that 230 gene products were upregulated by CBD+LPS and 236 transcripts by THC+LPS whereas 51 gene products were downregulated by CBD+LPS and 31 transcripts by THC+LPS (**Figure 1A**).

Our results also reveal that from the 5338 transcripts found to be differentially regulated by the various treatments (p ≤ 0.005), 680 gene probe sets were found to be upregulated by CBD alone and 58 gene products by THC alone. CBD had also a much larger effect compared to THC on the number of downregulated genes, 524 gene products were downregulated by CBD and 36 by THC (**Figure 1A**).

The groups of LPS-upregulated and downregulated genes that showed a change in expression of ≥2-fold (p≤0.005) in either direction were subjected to gene ontology (GO) analysis allowing functional annotation using the DAVID Bioinformatics Resources and the KEGG pathways (**Figure S1**). All major Biological Processes, Cellular Components and Molecular Functions within GO for the LPS-upregulated transcripts, were for the most part, genes associated with the immune and defense responses as well as apoptosis and cell death. KEGG database analysis included genes related to Toll-like receptor pathways and antigen processing as well as to the MAPK pathways. IPA global functional analysis of the LPS-upregulated and downregulated genes (≥2-fold; p≤ 0.005) confirmed the DAVID representation of genes within IPA-specific GO categories (**Figure S2**). IPA global functional analysis of the LPS-upregulated transcripts show gene products mainly implicated in cell-to-cell signaling, cellular movements, growth and proliferation, as well as cell death, immune response and signaling, including genes involved in the NF-κB pathway and in the activation of the liver X receptor/retinoic X receptor (LXR/RXR). The largest subsets of downregulated transcripts included genes known to be involved in the regulation of macromolecules and nucleotide metabolism, differentiation and development as well as regulation of gene expression and transcription (**Figures S1 and S2**).

A list of 183 LPS-upregulated genes (stimulated by 2-fold or more; p≤ 0.005) are presented in **Table S2**. This list includes LPS-upregulated genes that were (in most cases) significantly affected by CBD+LPS or by THC+LPS. They appear in the Table according to their categories and specific annotations based on their GO and IPA. The highly upregulated transcripts were related to inflammation, host defense and adaptive response. Inflammatory cytokines and chemokines together with their cognate receptors comprised a large group of more than 20 genes. Interferon related transcripts formed a significant population comprised of 12 genes. Based on GO and relevant literature references, genes were also classified into other functional categories including metabolic enzymes, membrane transport and secretion, kinases, phosphatases as well as transmembrane G-protein coupled receptors. The remainder of the LPS-upregulated genes was grouped according to their involvement in several other cellular processes such as apoptosis, proliferation and cell cycle progression, transcriptional and translational control as well as stress response. Additionally, we have identified a cluster of genes associated with regulation of extracellular matrix that are known to be involved in molecular recognition between cells, cell adhesion and migration. Genes implicated in cytoskeleton remodeling and control of cell motility and morphogenesis form a separate category.

The gene transcripts upregulated by LPS to the highest extent consist of *interleukin 1b* (*Il1b*; 135-fold), *colony stimulating factor 3 (Csf3*; 118-fold), *chemokine (C-X-C motif) ligand 2* (*Cxcl2*; also known as *MIP-2α*; 109-fold), *chemokine (C-X-C motif) ligand 10* (*Cxcl10*; 44-fold), *serum amyloid A3* (*Saa3*; 38-fold), *chemokine (C-C motif) receptor-like 2* (*Ccrl2*; 33-fold) and *CD69 antigen* (C*d69*; 30-fold); (**Table S2**).

Analysis of expression levels for the various transcripts affected by LPS showed that a much higher number of LPS-induced genes were significantly affected by pretreatment with CBD as compared to THC. From the 183 LPS-upregulated genes (by 2-fold or more; p≤0.005), 73 transcripts were inhibited by 30% or more, and 13 genes were repressed by more than 60% by CBD. These later genes are shown in **Table 1** and contain *Il-1b* (reduced by 81%), *dual specificity phosphatase 2* (*Dusp2*; by 78%), *chemokine (C-C motif) ligand 12* (*Ccl12*; by 77%), *chemokine (C-C motif) ligand 9* (*Ccl9*; by 76%), *endothelin 1* (*Edn1*; by 71%), *interferon beta 1* (*Ifnb1*; by 70%); *chemokine (C-C motif) ligand 7* (*Ccl7*; by 70%), *Cd69* by 69%, *formyl peptide receptor, related sequence 2* (*Fpr-rs2*; by 69%), *Il-1a* (by 68%), *interleukin 4 induced 1* (*Il4i1*; by 63%); *interleukin 27* (*Il27*; by 62%), *paired immunoglobin-like type 2 receptor alpha* (*Pilra*; by 62%), *matrix metalloproteinase 13* (*Mmp 13*; by 53%) and *chemokine (C-C motif) ligand 2* (*Ccl2*; by 52%).

**Table 1:** Repressive effect of CBD and THC on LPS-upregulated genes.

Gene Name	Description	Accession	Fold Change (versus untreated)					Effect on LPS	
*Proinflammatory cytokines*			LPS	CBD+LPS	THC+LPS	CBD	THC	CBD	THC
*Il1b*	interleukin 1 beta	NM_008361	135.3	26.4	97.7	1.0	1.3	**−81%**	**−28%**
*Il1a*	interleukin 1 alpha	NM_010554	22.9	7.3	18.1	0.9	1.2	**−68%**	**−21%**
*Il27*	interleukin 27	NM_145636	7.7	2.9	6.8	0.9	1.0	**−62%**	**−12%**
***Anti-inflammatory cytokines***									
*Il4i1*	interleukin 4 induced 1	NM_010215.3	10.0	3.7	8.1	1.0	0.9	**−63%**	**−19%**
***Proinflammatory chemokines***									
*Ccl12*	chemokine (C-C motif) ligand 12	NM_011331	9.7	2.3	7.3	0.6	1.3	**−77%**	**−25%**
*Ccl7*	chemokine (C-C motif) ligand 7	NM_013654	8.6	2.5	7.9	0.3	1.2	**−70%**	**−9%**
*Ccl9*	chemokine (C-C motif) ligand 9	NM_011338.2	4.9	1.2	4.7	0.5	1.2	**−76%**	**−3%**
*Ccl2 (MCP-1)*	chemokine (C-C motif) ligand 2	NM_011333.3	4.5	2.1	4.5	0.3	1.0	**−52%**	**0%**
***Interferon regulated***									
*Ifnb1* *	interferon beta 1	NM_010510.1	11.1	3.3	6.9	1.2	1.0	**−70%**	**−38%**
***Host defense and adaptive response***									
*Cd69* **	CD69 antigen	NM_001033122	29.9	9.2	27.5	1.0	1.5	**−69%**	**−8%**
*Edn1* **	endothelin 1	NM_010104.2	11.8	3.4	11.2	0.9	1.0	**−71%**	**−5%**
*Pilra* **	paired immunoglobin-like type 2 receptor alpha	NM_153510.3	6.3	2.4	3.9	0.7	1.5	**−62%**	**−38%**
***Phosphatases***									
*Dusp2*	dual specificity phosphatase 2	NM_010090	3.1	0.7	1.7	0.6	1.0	**−78%**	**−45%**
***Motility and morphogenesis***									
*Fpr-rs2* **	formyl peptide receptor, related sequence 2	NM_008039.2	11.7	3.6	8.9	0.9	1.6	**−69%**	**−24%**
***Adhesion and migration***									
*Mmp13* *	matrix metalloproteinase 13	NM_008607.1	17.4	8.1	19	0.8	1.2	**−53%**	**9%**

* genes related to proinflammatory signaling.

** genes related to inflammatory responses.

When CBD was applied without LPS stimulation, it did not have a significant effect on these genes, except for *Ccl7, Ccl2* and *Ccl9* which were downregulated by CBD by 71.4%, 71.4% and 50%, respectively. Careful evaluation of the list of genes reveals that the inhibitory effect of CBD on LPS-upregulated genes was concentrated mainly within gene categories related to inflammation. These included cytokines and chemokines, interferon regulated components of the immune response as well as molecules involved in host defense and adaptive immune response. The inhibitory effect of CBD on LPS-upregulated genes was less strong on other gene products such as those for regulatory molecules, kinases, phosphatases, metabolic enzymes, G protein-coupled receptors and molecules related to regulation of transcription.

THC inhibited the expression of only some of the LPS-upregulated genes and in most cases its effect was smaller than that of CBD: *Dusp2* was reduced by 45%; *Ifnb1*, by 38%; *Pilra*, by 38%; *IL-1b*, by 28%; *Ccl12*, by 25%; *Fpr-rs2*, by 24% and *IL-1a*, by 21%); (**Table 1**). The expression of genes downregulated exclusively by THC by more than 30% included only *haptoglobin* (*Hp*; reduced by 45%); (**Table S2**). Analyzing the relationship (using DAVID Bioinformatics, KEGG pathways and BioCarta pathway maps) between the list of genes affected by LPS and those downregulated when CBD or THC were added, we found that the downregulated genes are related to a number of inflammatory pathways including Toll-like receptor, Jak/STAT, IL-10 or IFNβ signaling pathways.

Interestingly, we found a significant number of LPS-upregulated genes whose expression was increased by CBD pretreatment. CBD enhanced the expression of 165 genes from the list of the ≥2-fold LPS-upregulated genes (**Table S2**). The expression of 28 of these genes was enhanced by more than 30%. These genes were present mainly within the categories of regulation of transcription, cell cycle and proliferation, apoptosis, membrane transport and secretion, phosphatases, cell adhesion as well as of host defense and adaptive responses. Among them, we would like to mention (**Table 2**) 15 genes whose expression was largely enhanced by CBD. These included *aquaporin 9* (*Aqp9*; upregulated by 877%), growth differentiation factor 15 (*Gdf15*; also known as macrophage inhibitory cytokine 1, *MIC-1*; by 592%), *lipocalin 2* (*Lcn2*; by 421%), *Cdkn2b/p15* (by 314%), *prostaglandin 1 receptor* (*Ptgir*; by 207%), *solute carrier family 7 member 11* (*Slc7a11*; by 157%), *sequestosome 1/p62* (*Sqstm1/p62*; by 117%), *Jun dimerization protein 2* (*Jundm2*; by 117%); *villin 2* (*Vil2*; by 110%), *dual specificity phosphatase 8* (*Dusp8*; by 107%), *nuclear protein 1/p8* (*Nupr1/p8*; by 107%), *mucolipin 2* (*Mcoln2/TRPML2*; by 104%), *Dusp1* (by 103%), *C-type (calcium dependent*, *carbohydrate recognition domain) lectin* (*Clecsf9*; by 59%) and *homocysteine-inducible*, *endoplasmic reticulum stress-inducible, ubiquitin-like domain member 1* (*Herpud1*; 37%). It is important to note that synergistic-like effect between CBD and LPS was observed regarding the expression of *Aqp9, Gdf15, Lcn2, Cdkn2b, Ptgir, Slc7a11* and *Clecsf9*.

**Table 2:** Enhanced effect of CBD and THC on LPS-upregulated genes.

Gene Name	Description	Accession	Fold Change (versus untreated)					Effect on LPS	
*Anti-inflammatory cytokines*			LPS	CBD+LPS	THC+LPS	CBD	THC	CBD	THC
*Gdf15*	growth differentiation factor 15	NM_011819.2	8.1	55.7	15.5	8.2	2.4	592%	92%
***Regulatory molecules***									
*Sqstm1/p62*	sequestosome 1	NM_011018	2.3	5.1	3.3	3.2	1.2	117%	40%
***Membrane transport and secretion***									
*Slc7a11*	solute carrier family 7 (cationic amino acid transporter, y+ system), member 11	NM_011990.1	8.0	20.5	10.1	7.1	1.7	157%	26%
*Aqp9*	aquaporin 9	NM_022026.2	3.1	30.3	4.8	14.3	2.0	877%	55%
*Mcoln2 (TRPML2)*	mucolipin 2	NM_026656.4	2.8	5.7	3.6	1.8	1.3	104%	28%
***Phosphatases***									
*Dusp1 (MKP-1)*	dual specificity phosphatase 1	NM_013642	2.5	5.1	2.9	2.8	1.1	103%	16%
*Dusp8*	dual specificity phosphatase 8	NM_008748	2.5	5.1	2.5	1.6	1.0	107%	0%
***G protein-coupled receptors***									
*Ptgir*	prostaglandin I receptor	NM_008967.1	3.8	11.7	4.3	5.4	1.3	207%	13%
***Apoptosis***									
*Lcn2*	lipocalin 2	NM_008491.1	13.3	69.1	10.9	2.5	2.5	421%	−18%
***Regulation of transcription***									
*Nupr1*	nuclear protein 1	NM_019738.1	4.3	8.8	5.4	7.9	2.0	107%	27%
*Jundm2*	Jun dimerization protein 2	NM_030887.2	3.0	6.5	4.1	3.0	1.5	117%	38%
***Cell cycle and proliferation***									
*Cdkn2b*	cyclin-dependent kinase inhibitor 2B (p15,inhibits CDK4)	NM_007670.2	2.2	9.1	3.1	2.9	1.1	314%	41%
***Stress response***									
*Herpud1*	homocysteine-inducible, endoplasmic reticulum stress-inducible, ubiquitin-like domain member 1	NM_022331.1	3.2	4.4	4.6	3.3	2.3	37%	42%
***Adhesion and migration***									
*Clecsf9* *	C-type (calcium dependent, carbohydrate recognition domain) lectin	NM_019948.2	20.3	32.3	26.5	2.3	1.7	59%	30%
*Vil2/Ezrin*	villin 2	NM_009510.2	2.6	5.4	2.9	1.6	1.0	110%	12%

* gene involved in proinflammatory signaling.

In contrast to CBD, a smaller number of LPS-upregulated genes showed increased expression as a result of treatment with THC+LPS (**Table 2**). These included *Gdf15* (enhanced by 92%), *Aqp9* (by 55%), *interleukin 15* (*Il15*; by 47%), *Herpud1* (by 42%), *Cdkn2b* (by 41%), *Sqstm1/p62* (by 40%), *Jundm2* (by38%) and *Clecsf9* (by 30%). From these transcripts, only two gene products, *Gdf15* and *Clecsf9*, were induced synergistically by the combination of THC+LPS. As for the remainder of THC sensitive genes, only in two cases we have seen enhancement of the LPS-upregulated mRNA expression levels by THC and not by CBD. These included *guanylate nucleotide binding protein 5* (*Gbp5*; 36%) and *myxovirus resistance 1* (*Mx1*; 33%); (**Table S2**).

As for the 145 transcripts that were downregulated by ≥2-fold (i.e., by 50% or more; p≤ 0.005) after LPS treatment, **Table S3** shows 82 gene products that were affected by CBD+LPS or by THC+LPS. The largest subsets of LPS-downregulated transcripts include genes known to be involved in cell cycle and proliferation, as well as in regulation of gene expression and transcription. Among the 33 genes that were downregulated by LPS treatment by ≥3-fold (i.e., by 75% or more; p≤0.005), the most affected genes included *Epstein-Barr virus induced gene 2/G protein-coupled receptor 183* (*Ebi2/Gpr183*; reduced by 89%), *kelch-like 6* (*Klhl6*; by 87.5%), *lymphoblastomic leukemia* (*Lyl1*; by 81.5%), *pleckstrin homology, sec7 and coiled-coil domains, binding protein* (*Pscdbp*; by 80.8%), *beta galactoside alpha 2,6 sialyltransferase 1* (*St6gal1*; by 78.7%), *sestrin 1* (*Sesn1*; by 77.3%), *ubiquitin specific protease 2, transcript variant 2* (*Usp2*; by 76.7%), *CD28 antigen* (*Cd28*; by 75.6%) and *c-mer proto-oncogene tyrosine kinase* (*Mertk*; by 75%); (**Table S3**).

From the 82 LPS-downregulated genes presented in **Table S3**, CBD pretreatment counteracted the reduction in mRNA levels of 16 genes (by 30% or more) and 2 were affected by more than 60%. These genes almost exclusively belong to the functional categories of cell adhesion and migration, motility and morphogenesis, regulation of transcription as well as of membrane transport and secretion. These genes (**Table 3**) include *neurophilin 1(Nrp1*; whose reduction was completely abolished), *neural precursor cell expressed developmentally down-regulated gene 9 (Nedd9*; reversed by 69%), *basic helix-loop-helix domain containing class B2 (Bhlhb2*; by 56%), *unc-5 homolog B (Unc5b*; by 53%), *Cd28* (by 49%), *SRY-box containing gene 4* (*Sox4*; by 42%) and *potassium channel, subfamily K, member 13* (*Kcnk13*; by 42%). Treatment with THC resulted in a much weaker attenuation of the LPS-induced downregulation of gene expression and only in the case of *Nrp1* exceeded 30%. DAVID Bioinformatics Resources and KEGG pathway analyses identify *Nrp1* and *Unc5b* as genes related to axon guidance and *Nedd9* as a gene related to adherens junction dynamics.

**Table 3:** Opposing effects of CBD and THC on LPS-downregulated genes.

Gene Name	Description	Accession	Fold Change (versus untreated)					Effect on LPS	
*Adhesion and migration*			LPS	CBD+LPS	THC+LPS	CBD	THC	CBD	THC
*Cd28* *	CD28 antigen	NM_007642.2	−4.1	−2.1	−3.3	1.3	−1.2	**−49%**	**−20%**
*Nrp1*	neuropilin 1	NM_008737.1	−3.4	0.0	−2.2	1.6	−1.1	**−100%**	**−33%**
*Unc5b*	unc-5 homolog B (C. elegans)	NM_029770.1	−2.9	1.4	−2.4	1.7	−1.4	**−53%**	**−15%**
***Motility and morphogenesis***									
*Nedd9*	neural precursor cell expressed, developmentally down-regulated gene 9	NM_017464.2	−2.8	−0.9	−2.3	−1.0	−1.4	**−69%**	**−20%**
***Membrane transport and secretion***									
*Kcnk13*	potassium channel, subfamily K, member 13	NM_146037.1	−2.2	−1.3	−1.6	−0.9	−0.9	**−42%**	**−27%**
***Regulation of transcription***									
*Sox4*	SRY-box containing gene 4	NM_009238.1	−3.1	−1.8	−2.8	2.1	0.9	**−42%**	**−10%**
*Bhlhb2*	basic helix-loop-helix domain containing, class B2	NM_011498.4	−2.5	−1.1	−1.9	1.0	1.0	**−56%**	**−24%**

* gene related to inflammatory responses.

In several cases, we have observed that treatment with CBD further potentiated the LPS-downregulation of some genes. These genes were spotted across almost all identified gene categories; however they were particularly concentrated within those related to cell cycle and proliferation, stress response, metabolic processes and phosphatases. Thus, CBD significantly enhanced the LPS-downregulation of a number of important genes including *cyclin D1* (*Ccnd1*; by additional 155%), *Pscdbp* (by 111%), *myocyte enhancer factor 2c* (*Mef2c*, by 95%), *Cxcl14* (by 93%), *paladin* (*Pald*; by 88%), *Ebi2/Gpr183* (by 82%), *Klhl6* (by 72%) and *RAS guanyl releasing protein 3 (Rasgrp3*, by 66%), *heat shock 70 kDa protein 5 binding protein 1* (*Hspa5bp1*, by 64%); *zinc finger protein 36, C3H type-like 1* (*Zfp 36l1*, by 61%), *carbohydrate sulfotransferase 10* (*Chst10*, by 59%) and *SH3-binding kinase* (*Sbk*, by 51%); (**Table 4**). As for THC, from the 75 genes that were downregulated when both THC and LPS were present, only two transcripts were markedly downregulated due to the THC treatment. These included *Pscdbp* reduced by 40% and *Chst10* by 38% compared with the levels obtained with LPS alone.

**Table 4:** Potentiated effect of CBD and THC on LPS-downregulated genes.

Gene Name	Description	Accession	Fold Change (versus untreated)					Effect on LPS	
*Stress response*			LPS	CBD+LPS	THC+LPS	CBD	THC	CBD	THC
*Sesn1*	sestrin 1	NM_001013370.2	−4.4	−6.3	−4.2	−1.7	−1.1	43%	−3%
*Hspa5bp1*	heat shock 70 kDa protein 5 binding protein 1	NM_133804.1	−3.6	−5.8	−4.1	−1.0	−1.5	64%	14%
***Proinflammatory chemokines***									
*Cxcl14*	chemokine (C-X-C motif) ligand 14	NM_019568.2	−1.1	−2.1	−1.2	−2.6	−1.4	93%	11%
***Metabolic enzymes***									
*Chst10*	carbohydrate sulfotransferase 10	NM_145142.1	−3.2	−5.0	−4.3	−2.2	−1.8	59%	38%
***Cell cycle and proliferation***									
*Klhl6*	kelch-like 6 (Drosophila)	NM_183390.1	−8.0	−13.7	−9.1	−1.9	−1.4	72%	13%
*Rasgrp3*	RAS guanyl releasing protein 3	NM_207246.2	−2.7	−4.5	−2.4	−1.4	−1.1	66%	−13%
*Ccnd1*	cyclin D1	NM_007631.2	−2.4	−6.0	−2.8	−2.9	−1.2	155%	20%
*Zfp36l1*	zinc finger protein 36, C3H type-like 1	NM_007564.2	−2.0	−3.2	−2.2	−1.1	−1.0	61%	9%
***Motility and morphogenesis***									
*Pscdbp* *	pleckstrin homology, Sec7 and coiled-coil domains, binding protein	NM_139200.4	−5.2	−11.0	−7.3	−2.1	−1.7	111%	40%
***Phosphatases***									
*Pald*	paladin	NM_013753.1	−3.0	−5.7	−3.3	−2.0	−1.2	88%	9%
***Kinases***									
*Sbk*	SH3-binding kinase	NM_145587.1	−2.2	−3.2	−2.3	−1.2	−1.2	51%	8%
***G protein-coupled receptors***									
*Ebi2*	Epstein-Barr virus induced gene 2/GprPR183	NM_183031.2	−9.1	−16.6	−10.6	−2.4	−1.3	82%	16%
***Regulation of transcription***									
*Mef2c*	myocyte enhancer factor 2C	NM_025282.1	−2.1	−4.1	−2.3	−2.1	−1.2	95%	11%

* gene related to inflammatory responses.

### Validation of microarray results

A large number of genes that were identified by microarray analysis as differentially regulated were subjected to validation by qPCR using *B2m* as a reference gene. Many of them were selected to be validated according to the distinct effects of cannabinoids on the LPS affected genes. The qPCR assays were repeated several times using at least 3 mRNA preparations from independent experiments. The results are expressed as fold change relative to control levels. We found that in almost all cases, there has been a very good agreement between the microarray and the qPCR data in terms of direction of change as well as of its magnitude (**Table 5**). For example, the qPCR data show that LPS upregulates the expression of the inflammatory chemokines *Ccl2, Ccl12* and *Cxcl10*, the transporters and channels *Aqp9, Mcoln2 (TRPML2)* and *Slc7a11*, the kinase inhibitor *Cdkn1a*, the phosphatases *Dusp1* and *Dusp2*, the regulatory molecule *Sqstm1/p62*, the G protein-coupled receptor *Ptgir* and the receptor *Tlr2*. The qPCR data also show that LPS downregulates the expression of the G protein-coupled receptors *Ebi2/Gpr183, Cnr2* and *Gpr55*, and the transcription factors *Pparg1* and *Pparg2*. In addition, in agreement with the microarray data, the stress response genes *Trib3* and *Hmox1* are upregulated by CBD+LPS but not by LPS alone or by THC+LPS.

**Table 5:** qCR validation.

Gene Name	Fold Change				
*Inflammatory chemokines*	LPS	CBD+LPS	THC+LPS	CBD	THC
Ccl2	5.2±1.5	2.8±0.3	5.7±1.3	0.5±0.08	1.2±0.1
Ccl12	10.7±2.5	3.8±0.4	5.8±1.2	0.8±0.2	0.9±0.1
Cxcl10	52.7±3.2	41.2±5.1	45.1±2.8	1.8±0.3	0.7±0.1
***Membrane transport and secretion***					
*Aqp9*	2.5±0.3	34.7±7.1	6.8±1.6	14.6±3.8	2.5±0.5
*Mcoln2 (TRPML2)*	3.9±0.3	5.3±0.1	4.0±0.6	1.5±0.8	1.6±0.5
*Slc7a11*	6.5±0.3	19.8±0.1	15.3±0.1	9.2±0.6	1.4±0.2
***Cell cycle and proliferation***					
*Cdkn1a/p21*	7.3±2.1	12.6±2.2	6.2±0.7	3.2±0.6	1.0±0.1
***Phosphatases***					
*Dusp1*	2.4±0.2	9.1±1.8	2.2±0.4	3.2±1.5	0.8±0.1
*Dusp2*	2.5±0.2	1.1±0.5	2.2±0.2	0.8±0.2	0.9±0.2
***Regulatory molecules***					
*Sqstm1/p62*	3.0±0.8	5.9±1.8	3.2±0.8	4.8±1.7	1.7±0.1
***G protein-coupled receptors***					
*Ebi2/Gpr183*	0.3±0.1	0.06±0.02	0.08±0.01	0.24±0.01	1.06±0.19
*Ptgir*	5.3±0.7	13.4±0.8	7.7±0.6	11.9±0.6	1.6±0.2
*GPR55*	0.19±0.04	0.15±0.03	0.29±0.07	0.83±0.06	1.0±0.2
*Cnr2*	0.1±0.01	0.1±0.02	0.15±0.02	0.9±0.1	1.05±0.15
***Host defense and adaptive response***					
*Tlr2*	4.1±0.9	2.6±0.3	3.2±0.6	1.1±0.1	1.1±0.2
***Stress response***					
*Trib 3*	0.6±0.1	15.2±0.4	1.6±0.2	15.7±0.3	3.6±0.9
*Hmox1*	0.84±0.04	3.6±0.9	0.9±0.2	3.9±1.2	2.1±0.2
***Transcription factors***					
*Pparg1*	0.14±0.01	0.27±0.06	0.12±0.03	1.4±0.3	1.27±0.01
*Pparg2*	0.15±0.02	0.06±0.01	0.35±0.06	1.6±0.2	1.0±0.1

### Network analysis and signaling pathways – Functional associations between the effects of cannabinoids and LPS

The differential mRNA expression patterns as revealed by the arrays of BV-2 cells treated with LPS alone or in combination with either CBD or THC, were analyzed using the Ingenuity System Database, software that includes the Ingenuity Knowledge Base (IKB) and the Global Molecular Network (GMN). These databases integrate published findings on biologically meaningful genetic or molecular gene/gene product interactions and identify functionally related gene networks.

**Figure 2:**
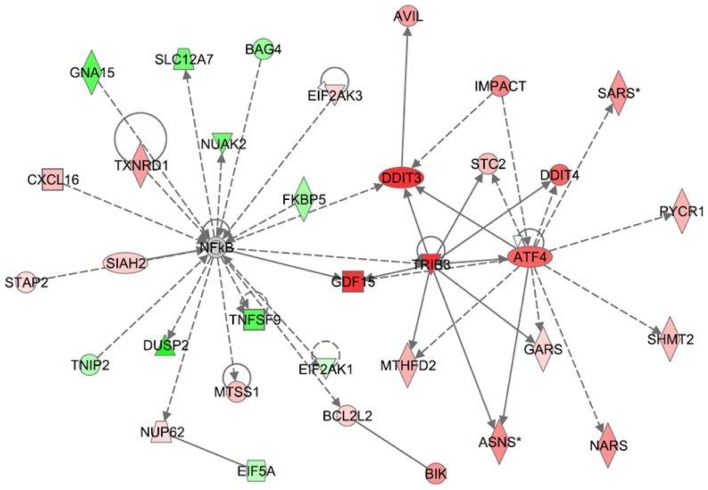
Ingenuity Interactome analysis of the effect of CBD on LPS-affected gene expression. Network analysis of the genes whose expression was affected by LPS+CBD vs LPS was performed using the Ingenuity software. Each network displays the genes/gene products as nodes (different shapes representing the functional classes of gene products) and the biological relationships between the nodes as lines. The length of each line reflects the amount of literature evidence supporting this node-to-node relationship. The color intensity of each node indicates the degree of upregulation (red) or downregulation (green) of the respective gene transcript. Genes in gray have been shown (in the literature) to interact with the colored gene products that appear in this scheme. **Network 1**: The NFκB-ATF4 interactome – Interaction between the CBD-upregulated *Trib3* and the attenuation of the NF-κB transcription factor (responsible for the transcription of many proinflammatory genes) shows the connection of this network to anti-inflammatory responses.

The differential gene expression values (*e.g.*; CBD+LPS versus LPS treatment or THC+LPS versus LPS) were entered into IPA to determine the most highly regulated networks of gene interactions and to highlight the biological processes that are relevant to each of the treatments. The IPA computation gives a score for each network according to the fit of the set of supplied affected genes that are also contained in the GMN (focus genes: network eligible molecules). The scores indicate the likelihood that “focus genes” belong to a network versus those that are obtained by chance. A score of >2 indicates a ≥99% confidence that a “focus gene” network was not generated by chance. According to the degree of interconnectedness among the molecules, a higher or lower IPA network score is assigned. Only networks with a score of 15 or higher, have been selected for further analysis. Networks describing the relationships between a subset of genes and their neighboring genes are presented in **Figures 2** **,** **3** **,** **4** for CBD and **Figures 5** **,** **6** **,** **7** for THC. Focus genes are denoted by red symbols for upregulated genes and green symbols indicate downregulated genes. Grey and open symbols are intermediate molecules, placed in the network by the Ingenuity software and shown in the literature to interact with genes in this dataset. Symbols representing the functional categories of the molecules are listed in the legend to **Figure 2**.

**Figure 3:**
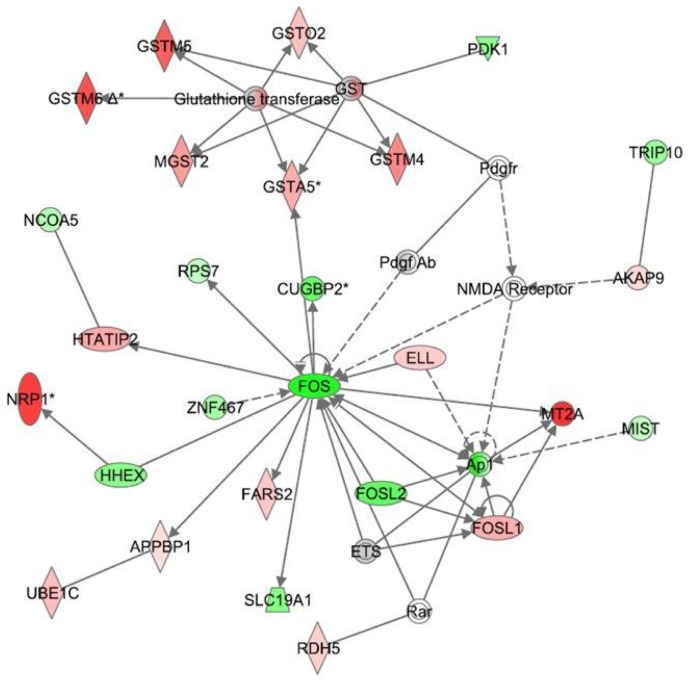
Ingenuity Interactome analysis of the effect of CBD on LPS-affected gene expression. **Network 2**: The FOS interactome – Interaction between the downregulated AP1 (a modulator of inflammatory mediated cellular functions) and the upregulated *FosL1* (a negative regulator of AP1) shows the connection of this network to CBD-anti-inflammatory responses Details are as indicated in Figure 2.

**Figure 4:**
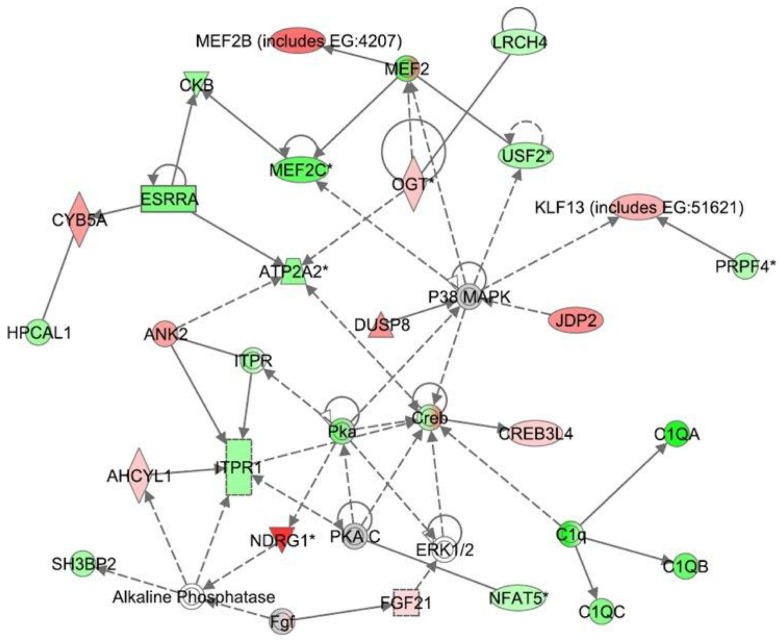
Ingenuity Interactome analysis of the effect of CBD on LPS-affected gene expression. **Network 3:** p38MAPK interactome Details are as indicated in Figure 2.

**Figure 5:**
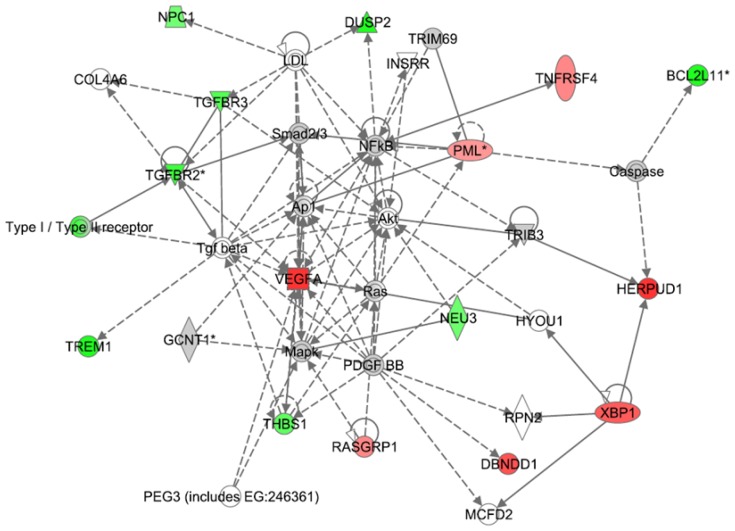
Ingenuity Interactome analysis of the effect of THC on LPS-affected gene expression. **Network 4**: The AP1-Akt-NFκB interactome Network analysis of the genes whose expression was affected by LPS+THC vs LPS was performed using the Ingenuity software. Details are as indicated in Figure 2.

**Figure 6:**
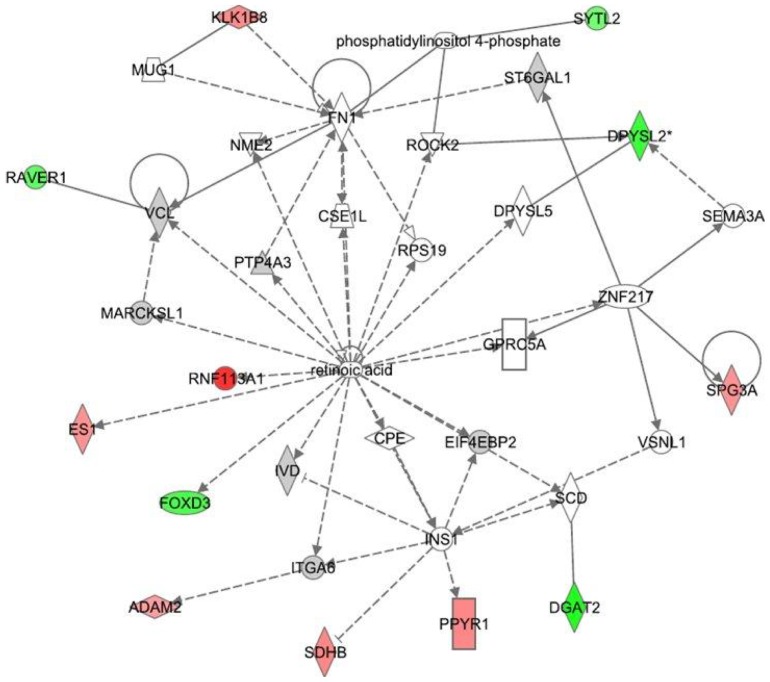
Ingenuity Interactome analysis of the effect of THC on LPS-affected gene expression. **Network 5:** The retinoic acid interactome Details are as indicated in Figure 2.

**Figure 7:**
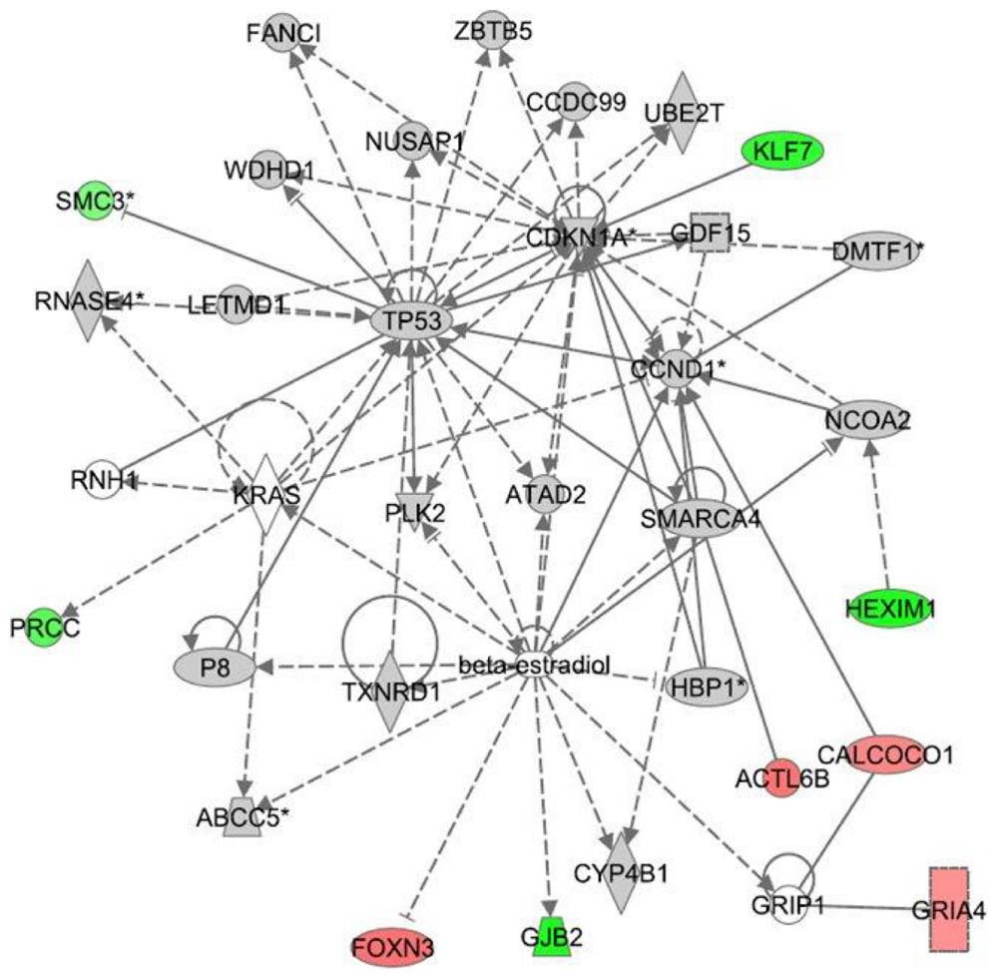
Ingenuity Interactome analysis of the effect of THC on LPS-affected gene expression. **Network 6:** TP53-Cdkn1A-beta estradiol interactome. Details are as indicated in Figure 2.

Interactome analysis between CBD+LPS- and LPS-associated gene networks are shown in **Figure 2** **–** **4** **. Network 1** (**Figure 2**) received the highest score (48) and contains 34 differentially regulated genes. This network has in its central position two transcription factors, ATF4 and NFκB, interconnecting several important canonical pathways. The later include Nrf2/ATF4-mediated oxidative stress response (including the highly upregulated *Trib3, Didit3/Chop/Gadd153, Asns, Gfd15*), aminoacyl-tRNA synthetases (including *Sars*, *Nars*, *Gars*), and ERK/MAPK and related phosphatases (such as *Dusp2* and *EIF2AK1*). In addition, 3 members of the Bcl-2 family, *Bik, Bcl2l2 and Bag4*, related to apoptotic processes were found to be present in the NFκB interactome. The Nrf2/ATF4-regulated transcripts were mainly upregulated (red symbols) whereas the genes downstream of NFκB were mostly downregulated (green symbols). The genes *Trib3* and *Gdf15* seem to serve as a point of interaction between these two families of gene transcripts. The ATF4 and NFκB networks are involved in cellular and molecular functions of cell death, protein synthesis and cancer [49], [50].

**Network 2** (**Figure 3**) with a score of 32 contains 27 genes. This network shows *Fos*, a member of the AP-1 transcription factor family, as a hub of connectivity, as it appears to play a central role possessing 15 edges (via direct connections). Two other transcripts belonging to the AP-1 superfamily, *FosL2/Fra-2* and *FosL1/Fra-1* (a gene whose product is known to decrease AP-1-dependent transcription) are also present in the *Fos* interactome. *FosL2/Fra-2* was found to be downregulated while *FosL1/Fra-1* is upregulated. A series of canonical pathways including those involved in glutathione and xenobiotic metabolism (*Mgst2, Gstm5, Gstm6, Gsto2, Gstm4, Gsta5, zinc metallothionein 2a* (*Mt2a*), *Znf467, folate transporter Slc19a1/Rfc1, Pdk1, Ube1c/Uba3*) as well as those involved in LPS/IL-1 mediated inhibition of either RXR or the peroxisome proliferator activated receptor (PPAR) signaling (*Rar, Rdh5, Ncoa5, Ap1, Fos, Ets*) were found to participate in this network. *Fos* directly interacts with *Gsta5* (from the glutathione-S-transferase family), known to belong together with cytochrome P450 enzymes, NAD(P) H:quinone oxido-reductase 1 (Nqo1) and aldehyde dehydrogenase 3 to a group of genes encoding phase 1 and phase 2 xenobiotic metabolizing enzymes [51]. Altogether, Network 2 is in correlation with a set of cellular and molecular functions which include amino acid-, vitamin- and mineral-metabolism and transport as well as cell signaling.

Network 3 (Figure 4) with a score of 28, includes 25 genes. This network presents p38MAPK in a central position and overlaps with canonical pathways for calcium signaling (*Nfat5*, *Itpr1, Pka, ATP2A2, Ank2, Ogt, Mef2b*), FGF pathway (*Fgf21, ERK1/2*), B cell signaling (*Mef2c, Itpr1, ERK1/2, Sh3bp2, Creb*) and p38MAPK signaling (*Dusp8, Mef2c, Creb, Nfat5, Esrra*). The IPA interactome analysis has linked the CBD-stimulated expression of *Dusp8* (via the negative modulation of p38MAPK) to the attenuation of LPS-mediated induction of *Nfat5*, a member of the inflammatory transcription family. In addition, 3 members of the complement component 1q (*C1qa, C1qb and C1qc*) that recognize antibody-bound pathogens and trigger the complement classical pathway, were found to be present in the p38MAPK interactome. This network seems to be related to functions of cell signaling, tissue morphology and neurological diseases.

The levels of changes in gene expression induced by combined treatment of LPS and THC (versus that induced by LPS alone) are smaller than those observed for the combined treatment of LPS with CBD (versus LPS alone). These LPS+THC affected genes encompassed three networks of specific molecular interactions. **Network 4** (**Figure 5**) with a score of 32 contains 16 differentially regulated focus molecules and presents the transcription factors AP-1, Akt and NFκB in its central positions. The gene products within the displayed network were found to be part of a number of canonical pathways. For example, the genes *X-box binding protein 1 (Xbp1)* and *Tgfbr2/3* are related to the glucocorticoid receptor signaling. The *progressive multifocal leukoencephalopathy* (*Pml*) is related to retinoic acid receptor (RAR) activation and PPAR signaling, and the *neuraminidase 3* (*Neu3*) to ceramide pathway and to p38MAPK. The gene *Trem1* is involved in immune response and *Vegfa* and *Thbs1* are related to the angiogenesis pathway. Altogether, this network shows that THC affects several biological processes including immune and lymphatic system development and function as well as tissue morphology.

THC also led to changes in the LPS effects on several other groups of genes. **Network 5** received a score of 23 and is populated with 12 genes, with retinoic acid as a transcription regulator positioned at its center (**Figure 6**). A number of canonical pathways related to axonal guidance (*Sema3a*, *Rock2, Dpysl2/Crmp2, Dpysl5/Cram, Spg3a/Atl1, Foxd3*), synaptic plasticity (*Raver1*), cell adhesion (*Adam2*), actin cytoskeleton (*Rock2, Foxd3*) as well as gene products involved in the activation of the nuclear receptor family of transcription factors LXR/RXR (*Es1, Rnf113a1*), were found to participate in this network. Cell to cell interaction as well as tissue development are the major biological processes related to this network.

Finally, **Network 6** with a score of 16 and 9 genes shows *Tp53, Cdkn1A* and beta-estradiol at the central nodes of the interactome (**Figure 7**). This network overlaps with the aryl hydrocarbon and glucocorticoid receptor signaling. Phosphatidylinositol 3-kinase (PI3K)/Akt (*Tp53/Trp53, Cdkn1A, Ccnd1*), p53 (*Tp53/Trp53, Prcc*, *Cdkn1A, Gdf15, Ccnd1, Smc3*), beta estradiol (*Foxn3, Gjb2, Hexim1, Gria4, Calcoco1*) as well as JAK/Stat (*Cdkn1A, Klf7*) signaling are affected. This network is correlated with functions of cell cycle control, reproductive malfunctions and cancer.

## Discussion

### Effect of cannabinoids on LPS-stimulated gene expression in BV-2 cells

This study addresses the effects of the two major cannabinoids present in cannabis, CBD and THC, on mRNA expression in LPS-stimulated BV-2 microglial cells. Our results show that pretreatments with CBD or THC differentially affect LPS-regulated gene transcription. The modulation of LPS-regulated gene expression by CBD was more pronounced as compared with THC. This result is in line with the more profound effects of CBD on mRNA regulation in surveillant (resting) microglial cells, as previously described by our group [11], [13].

Here we show that CBD, more than THC, suppresses the LPS-induction of many proinflammatory genes in selected functional categories (**Table 1** and **Table S2**) including cytokines (*e.g.*, *Il-1β, Il-1α, Il-27, Il-6*, *Il-18, Tnfrsf5/Cd40*) and chemokines (*e.g.*; *Ccl12, Ccl7, Ccl9* and *Ccl2*). Moreover, CBD also repressed the basal expression of *Ccl2, Ccl7* and *Ccl9*. Interestingly, Ccl2, Ccl7 and Ccl12 bind to the same receptor (CCR2), thus, showing binding promiscuity while they activate different signal transduction pathways in different cell populations. From these chemokines, Ccl2 plays a critical role in multiple sclerosis and in its murine model, experimental autoimmune encephalomyelitis (EAE). Several reports show that increased expression of Ccl2 in immune cells is closely associated with the clinical activity of EAE ([52] and references therein). Thus, the CBD downregulation of *Ccl2* mRNA is in line with the finding that CBD is ameliorating the EAE disease symptoms [12].

Another hallmark of inflammation is the increased expression of proinflammatory mediators like matrix metalloproteinases (Mmps). Here, we show that CBD downregulates the expression of the LPS-upregulated matrix metalloproteinase13 (*Mmp13*). MMP13 level and activity are enhanced in correlation with the degenerative changes in osteoarthritis cartilage and this molecule co-localizes with its specific type II collagen cleavage products. In agreement with this result, Malfait et al., [53] reported that in the murine collagen-induced arthritis (CIA) CBD treatment blocks the progression of the disease.

CD69 antigen is another mediator found to be involved in the CIA model of rheumatoid arthritis. This antigen is highly expressed in the leukocyte infiltrates of various chronic inflammatory diseases. In this regard, CIA disease severity is increased by antibody-induced blockade of the transforming growth factor β (TGF-β) in wild type but not in CD69^-/-^ mice, suggesting that CD69 is a negative modulator of autoimmune response and inflammatory reaction [54]. Our current results show that CBD downregulates the mRNA expressions of the LPS-upregulated *Cd69* antigen and of *Mmp13* suggesting a possible mechanism for the therapeutic activity of CBD in the murine CIA model.

The expression of several genes in the inflammatory cytokine functional category was found to be enhanced by CBD (and to a lower extent by THC). These include the LPS-upregulated expression of *Gdf15/MIC-1* and *Il-15*. From these genes, *Gdf15* mRNA expression is upregulated (8.2-fold) in surveillant/resting BV-2 cells by CBD (and 2.4-fold by THC) but synergistically upregulated by CBD+LPS (55.7-fold). Gdf15/MIC-1 is a member of the TGF-β superfamily that plays key roles in the regulation of cellular responses to stress signals, inflammation and tissue repair. Gdf15 mRNA expression was found to be upregulated in activated macrophages by secreted proinflammatory cytokines (including TNFα, IL-1β and IL-6, as well as IL-2 and the macrophage colony-stimulating factor) suggesting that Gdf15 may act through an autocrine loop as an inhibitory factor in the late phases of inflammation by suppressing inflammation through the inhibition of macrophage activation [55].

Other genes highly upregulated by CBD are *Lcn2* and *Aqp9*. Lcn2 has been implicated in many cellular processes, such as cell death/survival, cell migration/invasion, cell differentiation, inflammation and iron sequestration, including a role in the acute phase response ([56] and references therein). With regard to Aqp9, we have previously shown that CBD upregulates *Aqp9* mRNA in BV-2 cells (14-fold; [13]). Moreover, CBD synergistically increases (by 877%) the LPS-upregulated *Aqp9* (3.1-fold). Aqp9 participates in the transport of small solutes (such as glycerol, lactate and urea) and takes part in osmotic swelling induced by apoptotic stimuli [57].

Two major kinase-mediated signaling pathways are activated following TLR4 stimulation by LPS. These include the MAPKs and IκB kinase complexes, which lead to activation of AP-1 and NF-κB transcription factors, respectively. Here we show that LPS-upregulated expression of *Dusp1* (MKP-1) was significantly upregulated by CBD (5.1-fold) but not by THC. An increase in the expression of *Dusp1* was also observed following incubation of resting BV-2 cells with CBD, but not with THC. A similar CBD-mediated regulation of mRNA level was observed for *Dusp8*, another negative regulator of MAPKs. The IPA interactome analysis has linked this CBD-stimulated expression of *Dusp8*, via negative modulation of p38 MAPK, to the attenuation of LPS-mediated induction of NFAT5, a member of the inflammatory transcription complex. On the other hand, the LPS-stimulated expression of *Dusp2* (*Pac-1*), a key positive regulator of the inflammatory MAPKs signaling [58], was significantly repressed by CBD as well as by THC. Moreover, CBD but not THC decreased basal levels of *Dusp2/Pac-1* mRNA in resting BV-2 cells.

IPA and interactome analysis indicate that pretreatment with CBD, but less so with THC, result in attenuation of LPS-stimulated activation of NF-κB and its dependent gene transcription pathways. Indeed, our previous data show that CBD decreases the activity of the NFκB signaling pathway in BV-2 microglial cells via the partial reversal of the LPS-induced degradation of IRAK-1 intermediate kinase, thus reversing IκB degradation and reducing NF-κB p65 subunit phosphorylation. This effect of CBD in decreasing NF-κB signaling activity is in line with the CBD-induced diminished transcription of NF-κB-dependent genes, *e.g.*, IL-1β and IL-6 [11].

We and others previously reported that BV-2 cells express CB_1_, CB_2_, GPR55, GPR18 and TRPV2, G protein-coupled receptors and a channel that are known to interact with cannabinoids [20], [30], [31], [59]–[61]. Using gene array analysis [13], [31], we have observed that the relative levels of *CB_1_, CB_2_, GPR18* and *TRPV2* as well as of the fatty acid amide hydrolase (*FAAH*; known to cleave the endocannabinoid anandamide) gene transcripts were not significantly affected by the cannabinoid treatments and their levels did not exceed the 2-fold induction or 50% reduction by either CBD or THC treatment. On the other hand, we show here that LPS markedly downregulates *CB_2_* and *GPR55* and that this downregulation is not affected by either CBD or THC pretreatment. This result is in agreement with our previous report showing that LPS markedly downregulates *CB_2_* and *GPR55* mRNAs in BV-2 microglial cells and in microglial primary cultures [59].

### Network analysis and signaling pathways

A relationship between CBD-mediated oxidative stress response and glutathione depletion was previously reported ([13] and references therein). More recently, we showed that CBD-specific gene expression profile in BV-2 cells displays changes normally occurring under either nutrient limiting conditions or proteasome inhibition, and that are attributed to activation of GCN2/eIF2α/p8/ATF4/CHOP-Trib3 pathway leading to autophagy as well as to apoptotic cell death [13]. The *Trib3* gene product seem to be of high importance to the CBD effect due to its ability to serve as a master regulator of an array of pathways including AP-1 [62], ER stress [63], Akt/PKB [64] and NF-κB [65]. *Trib3* expression is significantly upregulated by CBD (16-fold) as well as by THC (3.4-fold) [13] and as observed here, remains upregulated after LPS treatment (CBD + LPS, 14-fold; THC + LPS, 2.3-fold). According to these gene array studies and the qPCR results, LPS by itself does not significantly affect the expression of *Trib3* mRNA. IPA interactome analysis of the microarrays data reveals an interaction between the CBD-upregulated *Trib3* and the NF-κB transcription factor pathway (as shown in **Figure 2**). This interaction seems to be responsible for the attenuation by CBD of the transcription of many proinflammatory genes. There are several indications suggesting interaction between these two pathways. First, a direct interaction between p65/RelA and Trib3 protein which induces inhibition of PKA dependent p65 phosphorylation, was described [65]. Second, Trib3 protein can negatively regulate the serine-threonine kinase Akt/PKB [64], a downstream effector of PI3K that has been implicated in the potentiation of NF-κB-induced transcription of proinflammatory mediators [66]. This negative regulation of Akt activity by the highly induced *Trib3* gene product could point to the mechanism for the CBD-mediated regulation of LPS-stimulated gene expression. Indeed, the effect of CBD treatment on a number of LPS-stimulated genes as reported here is reminiscent of the effects described for the PI3K inhibitor (LY294002) and for the NFκB inhibitor (BAY 11-7082) in the murine macrophage cell line RAW264.7 activated with LPS [67]. Both PI3K and NFκB signaling pathways exert important roles in gene expression in response to LPS, but they are not overlapping. Specifically, treatment with CBD repressed a number of typical proinflammatory genes (*e.g.; CD40/Tnfrsf5, Tlr2* and *Ccl2/Mcp-1*) stimulated by LPS, which are known to be NFκB dependent and of other genes including *Csf3, Il-1β, Il-1α* and *Cox2/Ptgs2*, which are under the control of both PI3K and NFκB pathways.

Finally, Trib3 was documented to interfere with the inflammatory MAPK signaling via direct interaction with MEK-1 and MKK7 leading to attenuation of AP-1 mediated transcriptional activity in cancer HeLa cells [62]. AP-1 is a transcription factor involved in the regulation of inflammation-mediated cellular functions and has been shown to be inhibited by Nrf2-activating agents [68]. Indeed, our IPA network analysis indicates that the observed decrease in mRNA levels for a number of genes is probably related to a reduction in AP-1 dependent transcription. Additionally, according to these IPA results, this repression is reinforced by combined treatments of CBD and LPS as observed by the induction of *FosL1* gene product, another negative regulator of AP-1 (see **Figure 3**).

Trib3 has been shown to downregulate PPARγ transcription and serve as a potent negative regulator of adipocyte differentiation [69] and PPARγ is a molecular target for CBD that could be involved in mediating transcriptional effects in BV-2 microglial cells. Indeed, CBD has been shown to bind to PPARγ *in vitro* as well as to activate its transcriptional activity in 3T3L1 fibroblast and in HEK293 transfected cells [70]. In addition, Necela *et al*., [71] described a regulatory feedback loop in which PPARγ represses NF-κB-mediated inflammatory signaling in unstimulated macrophages. Moreover, they show that upon activation of TLR4 in LPS-stimulated macrophages, NF-κB drives down PPARγ expression. These results are in agreement with our results showing that LPS highly downregulates the expression of *Pparg1* and *Pparg2* (**Table 5**) in BV-2 cells.

The profiles of CBD-induced gene expression with either resting or LPS-activated BV-2 cells, show that CBD stimulates the transcription of several anabolic genes encoding amino acid biosynthetic enzymes, amino acid transporters and aminoacyl-tRNA synthetases known to be activated by ATF4, a basic leucine zipper transcription factor, that is increased when cultured cells are deprived of amino acids or subjected to endoplasmic reticulum (ER) stress ([13] and references therein). The divergent types of stress (nutrient deficiency, ER stress, hypoxia and oxidative stress) converge on a single event—phosphorylation of the translation initiation factor eIF2α, resulting in a general translational pause followed by selective increase in ATF4 mRNA translation and subsequent stimulation of expression of ATF4 target genes [72]. Many of the CBD-affected transcripts (*e.g., Herpud1, Hmox1*, *Nqo1*, *Gclm, Gstmu1, Gstmu6* upregulated by CBD and by CBD+LPS treatments) are indeed classified as Nrf2-mediated oxidative stress response genes, including enzymes involved in the biosynthesis of glutathione [13]. Thus, the observed CBD-mediated induction of ATF4-dependent anabolic genes may serve to replenish the amino acids reduced during the elevated turnover of GSH (cysteine, glutaConceived and designed the experiments:mate and glycine). The mechanism underlying CBD action presumably engages generation of ROS which in turn depletes intracellular GSH. Perturbations in redox tone and GSH levels activate the “phase 2 response”, a mechanism used by cells to mitigate oxidative stress [73]. As we have previously shown, many of the “phase 2” gene products are significantly upregulated by CBD [13].

Our present results show that CBD, and less so THC, have immunosuppressive and protective activities that are reminiscent of other clinically applied drugs such as glucocorticoids (GCs; [74]), rexinoids and synthetic triterpenoids [75]. GCs are immunomodulatory agents known to act as suppressive and protective mediators against inflammation. GCs are known to clear antigens by stimulating cell trafficking as well as scavenger systems and matrix metalloproteinases while they stop cellular immune responses by inhibiting antigen presentation and T cell activation [74]. Synthetic oleanane triterpenoids (SO) were shown to be highly effective (much more than the natural triterpenoids) in many *in vivo* models in the prevention and treatment of cancer and other diseases with an inflammatory component. Molecular targets of SO include KEAP1 (the inhibitor of the transcription factor Nrf2), PPARγ, IκB kinase, TGF-β signaling and STAT signaling [75]. SO are among the most potent known inducers of the phase 2 response both *in vivo* and *in vitro* and affect the expression of several key cell cycle proteins (*e.g.*, cyclin D1, Cdkn1a/p21 and caveolin 1). In some cancer cells, SO signal through PPARγ to inhibit proliferation. The rexinoids bind almost exclusively to the RXRs and are involved in regulation of development, cell proliferation, differentiation and apoptosis [76]. Because RXRs heterodimerize with other receptors (such as RARs, vitamin D receptor, thyroid hormone receptor, PPARα, PPARγ and LXRs), rexinoids modulate the actions of many steroid-like molecules that control metabolism and cellular energetics. In view of these results, triterpenoids and rexinoids are defined as multifunctional drugs. Their targets are either regulatory proteins that control the activity of transcription factors (triterpenoids) or transcription factors themselves (rexinoids). These complex modulatory activities exerted by GCs, rexinoids and SO display a panorama of effects that closely resembles the complex actions of CBD.

## Conclusions

We found that CBD induces robust responses related to oxidative stress and inflammation and that CBD, and to a lesser extent THC, have immunosuppressive and protective activities. The anti-inflammatory effects are mediated by mainly downregulating the expression of proinflammatory genes but also by upregulating anti-inflammatory mediators. The impact of pretreatment with CBD on LPS-stimulated gene expression was much greater than that of THC.

We show here, that CBD highly upregulates genes encoding negative regulators of NF-κB and AP-1 transcriptional activities (*e.g.; Trib3, Dusp1*). CBD response to oxidative stress and inflammation is controlled by Nrf2 and ATF4 transcription factors and seem to involve the Nrf2-Hmox1 and the Nrf2/ATF4 systems. The central role of phase 2-response genes and the ATF4-dependent *Trib3* induction offer an explanatory mechanism by which CBD (but less so THC) lowers the LPS-induced activation of microglial cells and leads to attenuation of transcription of many proinflammatory genes.

CBD and THC effects are also found to be linked to new targets, notably the MAPK pathway (*Dusp1/Mkp-1, Dusp8* and *Dusp2/Pac-1*), the JAK/STAT regulatory molecules (*Socs3, Cish, Stat1*) and the cell cycle related genes (*Cdkn2b/p15, Gadd45a, Cdkn1a/p21*).

All together, our results show that CBD and to a lesser extent THC, are potent modulators of microglial activation and affect several signaling pathways and related networks including induction of cellular stress responses that underlies their high immunosuppressant activity.

## Supporting Information

Figure S1 **Functional and pathway annotations according to DAVID Bioinformatics Resources of affected gene products following LPS treatment.** Gene Ontology (GO) analysis was performed separately for upregulated (red) and downregulated (green) genes, to identify functional categories and cluster of genes significantly affected by LPS treatment. The GO terms were arbitrarily chosen at various levels within the ontology to avoid redundant allocation. Categories shown are significantly represented at *p*≤0.05. The bottom pie charts represent the cellular pathways according to KEGG database.
(TIF)

Figure S2 **Distribution of differentially expressed genes by global functional annotation and pathway association according to IPA analysis.** IPA global functional and pathway analysis was used to examine the enriched functional classes of upregulated (warm colors) and downregulated (cold colors) gene transcripts among the LPS-stimulated genes. The *y* axis shows the top 8 most representative high-level functions and canonical pathways associated with genes regulated in LPS-treated BV-2 cells. The x axis displays the mean p-value for each associated high-level function and canonical pathway in a -log scale. Increasing value of −log (significance) indicates increased confidence for each category. The vertical gray line in each plot indicates *p* = 0.05.
(TIF)

Table S1 **Primer sequences for qPCR.** ^a^ Amplicon length in base pairs; ^b^ Genbank accession number of cDNA and corresponding gene, available at http://www.ncbi.nlm.nih.gov/http://www.ncbi.nlm.nih.gov/; ^c^ FW, forward primer; RV, reverse primer
(DOC)

Table S2 **Effect of CBD and THC on LPS-upregulated genes.** Some genes were validated by qPCR, using β2-microglobulin mRNA as a reference gene.
(XLS)

Table S3 **Effect of CBD and THC on LPS-downregulated genes.** Some genes were validated by qPCR, using β2-microglobulin mRNA as a reference gene.
(XLS)

## References

[1] Kogan NM, Mechoulam R (2007) Cannabinoids in health and disease. Dialogues Clin Neurosci 9: 413–430. 18286801 10.31887/DCNS.2007.9.4/nkogan PMC3202504

[2] Hill AJ, Williams CM, Whalley BJ, Stephens GJ (2012) Phytocannabinoids as novel therapeutic agents in CNS disorders. Pharmacology & Therapeutics 133: 79–97. 21924288 10.1016/j.pharmthera.2011.09.002

[3] Pertwee RG (2002) Cannabinoids and multiple sclerosis. Pharmacol Ther 95: 165–174. 12182963 10.1016/s0163-7258(02)00255-3

[4] Guzman M (2003) Cannabinoids: potential anticancer agents. Nat Rev Cancer 3: 745–755. 14570037 10.1038/nrc1188

[5] Di Marzo V, De Petrocellis L (2006) Plant, synthetic and endogenous cannabinoids in medicine. Ann Rev Med 57: 553–574. 16409166 10.1146/annurev.med.57.011205.135648

[6] Gowran A, Noonan J, Campbell VA (2011) The multiplicity of action of cannabinoids: Implications for treating neurodegeneration. CNS Neuroscience & Therapeutics 17: 637–644. 20875047 10.1111/j.1755-5949.2010.00195.x PMC6493861

[7] Cabral GA, Staab A (2005) Effects on the immune system. Handb Exp Pharmacol 168: 385–423. 10.1007/3-540-26573-2_13 16596782

[8] Klein TW, Cabral GA (2006) Cannabinoid-induced immune suppression and modulation of antigen-presenting cells. J Neuroimmune Pharmacol 1: 50–64. 18040791 10.1007/s11481-005-9007-x

[9] Ashton JC (2007) Cannabinoids for the treatment of inflammation. Curr Opin Investig Drugs 8: 373–384. 17520866

[10] Rieder SA, Chauhan A, Singh U, Nagarkatti M, Nagarkatti P (2010) Cannabinoid-induced apoptosis in immune cells as a pathway to immunosuppression. Immunobiology 215: 598–605. 19457575 10.1016/j.imbio.2009.04.001 PMC3005548

[11] Kozela E, Pietr M, Juknat A, Rimmerman N, Levy R, et al (2010) Cannabinoids Δ^9^ -tetrahydrocannabinol and cannabidiol differentially inhibit the LPS-activated NF-κB and IFNβ/STAT proinflammatory pathways in BV-2 microglial cells. J Biol Chem 285: 1616–1625. 19910459 10.1074/jbc.M109.069294 PMC2804319

[12] Kozela E, Lev N, Kaushansky N, Eilam R, Rimmerman N, et al (2011) Cannabidiol inhibits pathogenic T-cells, decreases spinal microglial activation and ameliorates multiple sclerosis-like disease in mice. Brit J Pharmacol 163: 1507–1519. 21449980 10.1111/j.1476-5381.2011.01379.x PMC3165959

[13] Juknat A, Pietr M, Kozela E, Rimmerman N, Levy R, et al (2012) Differential transcriptional profiles mediated by exposure to the cannabinoids cannabidiol and Δ^9^-tetrahydrocannabinol in BV-2 microglial cells. Brit J Pharmacol 165: 2512–2528. 21542829 10.1111/j.1476-5381.2011.01461.x PMC3423229

[14] Juknat A, Rimmerman N, Levy R, Vogel Z, Kozela E (2012) Cannabidiol affects the expression of genes involved in zinc homeostasis in BV-2 microglial cells. Neurochem Int 61: 923–930. 22178458 10.1016/j.neuint.2011.12.002

[15] van der Stelt M, Di Marzo V (2005) Cannabinoid receptors and their role in neuroprotection. Neuromolecular Med 7: 37–50. 16052037 10.1385/NMM:7:1-2:037

[16] Velasco G, Sánchez C, Guzmán M (2012) Towards the use of cannabinoids as antitumor agents. Nature Rev Cancer 12: 436–444. 22555283 10.1038/nrc3247

[17] Wu HY, Goble K, Mecha M, Wang CC, Huang CH, et al (2012) Cannabidiol-induced apoptosis in murine microglial cells through lipid raft. Glia 60: 1182–1190. 22535572 10.1002/glia.22345

[18] Cabral GA, Raborn ES, Griffin L, Dennis J, Marciano-Cabral F (2008) CB2 receptors in the brain: role in central immune function. Brit J Pharmacol 153: 240–251. 18037916 10.1038/sj.bjp.0707584 PMC2219530

[19] Pertwee RG, Howlett AC, Abood ME, Alexander SPH, Di Marzo V, et al (2010) International Union of Basic and Clinical Pharmacology. LXXIX. Cannabinoid Receptors and Their Ligands: Beyond CB1 and CB2. Pharmacol Rev 62: 588–631. 21079038 10.1124/pr.110.003004 PMC2993256

[20] Stella N (2010) Cannabinoid and cannabinoid-like receptors in microglia, astrocytes and astrocytomas. Glia 58: 1017–1030. 20468046 10.1002/glia.20983 PMC2919281

[21] Rhee MH, Vogel Z, Barg J, Bayewitch M, Levy R, et al (1997) Cannabinol derivatives: binding to cannabinoid receptors and inhibition of adenylylcyclase. J Med Chem 40: 3228–3233. 9379442 10.1021/jm970126f

[22] Cabral GA, Griffin-Thomas L (2009) Emerging role of the cannabinoid receptor CB2 in immune regulation: therapeutic prospects for neuroinflammation. Expert Rev Mol Med 11: e3. 19152719 10.1017/S1462399409000957 PMC2768535

[23] Mechoulam R, Peters M, Murillo-Rodriguez E, Hanus LO (2007) Cannabidiol-recent advances. Chem Biodivers 4: 1678–1692. 17712814 10.1002/cbdv.200790147

[24] Scuderi C, De Filippis D, Iuvone T, Blasio A, Steardo A, et al (2009) Cannabidiol in Medicine: a review of its therapeutic potential in CNS disorders. Phytother Res 23: 597–602. 18844286 10.1002/ptr.2625

[25] Booz GW (2011) Cannabidiol as an emergent therapeutic strategy for lessening the impact of inflammation on oxidative stress. Free Radical Biology & Medicine 51: 1054–1061. 21238581 10.1016/j.freeradbiomed.2011.01.007 PMC3085542

[26] Fernandez-Ruiz J, Sagredo O, Pazos MR, Garcia C, Pertwee R, et al (2013) Cannabidiol for neurodegenerative disorders: Important new clinical applications for this phytocannabinoid? Brit J Clin Pharmacol 75: 323–333. 22625422 10.1111/j.1365-2125.2012.04341.x PMC3579248

[27] Mecha M, Torrao AS, Mestre L, Carrillo-Salinas FJ, Mechoulam R, et al (2012) Cannabidiol protects oligodendrocyte progenitor cells from inflammation-induced apoptosis by attenuating endoplasmic reticulum stress. Cell Death and Disease 3: e331. 22739983 10.1038/cddis.2012.71 PMC3388241

[28] Pazos MR, Cinquina V, Gómez A, Layunta R, Santos M, et al (2012) Cannabidiol administration after hypoxia-ischemia to newborn rats reduces long-term brain injury and restores neurobehavioral function. Neuropharmacology 63: 776–783. 22659086 10.1016/j.neuropharm.2012.05.034

[29] Izzo AA, Borrelli F, Capasso R, Di Marzo V, Mechoulam R (2009) Non-psychotropic plant cannabinoids: new therapeutic opportunities from an ancient herb. Trends Pharmacol Sci 30: 515–527. 19729208 10.1016/j.tips.2009.07.006

[30] Rimmerman N, Kozela E, Levy R, Vogel Z, Juknat A (2013) Cannabinoid signaling through non CB1/non CB2 GPCR targets in microglia. In: Abood ME, Sorensen RG, Stella N, editors.endoCANNABINOIDS.Actions at non-CB1/CB2 cannabinoid receptors.Series: The Receptors, Vol. 24, pp.143–171 .Springer Verlag, New York.

[31] Rimmerman N, Juknat A, Kozela E, Levy R, Bradshaw HB, et al (2011) The non-psychoactive plant cannabinoid, cannabidiol affects cholesterol metabolism-related genes in microglial cells. Cell Mol Neurobiol 31: 921–930. 21533611 10.1007/s10571-011-9692-3 PMC11498456

[32] Srivastava MD, Srivastava BI, Brouhard B (1998) Delta9 tetrahydrocannabinol and cannabidiol alter cytokine production by human immune cells. Immunopharmacology 40: 179–185. 9858061 10.1016/s0162-3109(98)00041-1

[33] Cabral GA, Dove Pettit DA (1998) Drugs and immunity: cannabinoids and their role in decreased resistance to infectious disease. J Neuroimmunol 83: 116–123. 9610679 10.1016/s0165-5728(97)00227-0

[34] Klein TW, Newton C, Larsen K, Chou J, Perkins I, et al (2004) Cannabinoid receptors and T helper cells. J Neuroimmunol 147: 91–94. 14741435 10.1016/j.jneuroim.2003.10.019

[35] Puffenbarger RA, Boothe AC, Cabral GA (2000) Cannabinoids inhibit LPS-inducible cytokine mRNA expression in rat microglial cells. Glia 29: 58–69. 10594923

[36] Eljaschewitsch E, Witting A, Mawrin C, Lee T, Schmidt PM, et al (2006) The endocannabinoid anandamide protects neurons during CNS inflammation by induction of MKP-1 in microglial cells. Neuron 49: 67–79. 16387640 10.1016/j.neuron.2005.11.027

[37] Carrier EJ, Auchampach JA, Hillard CJ (2006) Inhibition of an equilibrative nucleoside transporter by cannabidiol: a mechanism of cannabinoid immunosuppression. Proc Natl Acad Sci USA 103: 7895–7900. 16672367 10.1073/pnas.0511232103 PMC1472541

[38] O′Sullivan SE (2007) Cannabinoids go nuclear: evidence for activation of peroxisome proliferator-activated receptors. Br J Pharmacol 152: 576–582. 17704824 10.1038/sj.bjp.0707423 PMC2190029

[39] Esposito G, Scuderi C, Valenza M, Togna GI, Latina V, et al (2011) Cannabidiol reduces Aβ-induced neuroinflammation and promotes hippocampal neurogenesis through PPARγ involvement. PLOS One 6: e28668. 22163051 10.1371/journal.pone.0028668 PMC3230631

[40] De Petrocellis L, Di Marzo V (2010) Non-CB_1_, non-CB_2_ receptors for endocannabinoids, plant cannabinoids and synthetic cannabimimetics: focus on G-protein coupled receptors and transient receptor potential channels. J Neuroimmune Pharmacol 5: 103–121. 19847654 10.1007/s11481-009-9177-z

[41] Graeber MB, Streit WJ (2010) Microglia: biology and pathology. Acta Neuropathol 119: 89–105. 20012873 10.1007/s00401-009-0622-0

[42] Saijo K, Glass CK (2011) Microglial cell origin and phenotypes in health and disease. Nature Rev Immunol 11: 775–787. 22025055 10.1038/nri3086

[43] Schwartz M, Shechter R (2010) Systemic inflammatory cells fight off neurodegenerative disease. Nat Rev Neurol 6: 405–410. 20531383 10.1038/nrneurol.2010.71

[44] Lund S, Christensen KV, Hedtjarn M, Mortensen AL, Hagberg H, et al (2006) The dynamics of the LPS triggered inflammatory response of murine microglia under different culture and *in vivo* conditions. J Neuroimmunol 180: 71–87. 16996144 10.1016/j.jneuroim.2006.07.007

[45] Thomas DM, Francescutti-Verbeem DM, Kuhn DM (2006) Gene expression profile of activated microglia under conditions associated with dopamine neuronal damage. Faseb J 20: 515–517. 16384912 10.1096/fj.05-4873fje

[46] Calvano SE, Xiao W, Richards DR, Felciano RM, Baker HV, et al (2005) A network-based analysis of systemic inflammation in humans. Nature 437: 1032–1037. 16136080 10.1038/nature03985

[47] Ashburner M, Ball CA, Blake JA, Botstein D, Butler H, et al (2000) Gene ontology: tool for the unification of biology. Nat Genet. 25: 25–29. 10.1038/75556 PMC3037419 10802651

[48] Huang DW, Sherman BT, Lempicki RA (2009) Systematic and integrative analysis of large gene lists using DAVID Bioinformatics Resources. Nature Protoc 4: 44–57. 19131956 10.1038/nprot.2008.211

[49] Baird TD, Wek RC (2012) Eukaryotic initiation factor 2 phosphorylation and translational control in metabolism. Adv Nutr 3: 307–321. 22585904 10.3945/an.112.002113 PMC3649462

[50] Ben-Neriah Y, Karin M (2011) Inflammation meets cancer, with NF-κB as the matchmaker. Nature Immunol 12: 715–723. 21772280 10.1038/ni.2060

[51] Dietrich C, Kaina B (2010) The aryl hydrocarbon receptor (AhR) in the regulation of cell-cell contact and tumor growth. Carcinogenesis 31: 1319–1328. 20106901 10.1093/carcin/bgq028 PMC6276890

[52] Banisor I, Leist TP, Kalman B (2005) Involvement of β-chemokines in the development of inflammatory demyelination. J Neuroinflammation 2: 7. 15730561 10.1186/1742-2094-2-7 PMC554759

[53] Malfait AM, Gallily R, Sumariwalla PF, Malik AS, Andreakos E, et al (2000) The nonpsychoactive cannabis constituent cannabidiol is an oral anti-arthritic therapeutic in murine collagen-induced arthritis. PNAS 97: 9561–9566. 10920191 10.1073/pnas.160105897 PMC16904

[54] Sancho D, Gómez M, Viedma F, Esplugues E, Gordón-Alonso M, et al (2003) CD69 downregulates autoimmune reactivity through active transforming growth factor-β production in collagen-induced arthritis. J Clin Invest 112: 872–882. 12975472 10.1172/JCI19112 PMC193672

[55] Bootcov MR, Bauskin AR, Valenzuela SM, Moore AG, Bansal M, et al (1997) MIC-1, a novel macrophage inhibitory cytokine, is a divergent member of the TGF-beta superfamily. Proc Natl Acad Sci USA 94: 11514–11519. 9326641 10.1073/pnas.94.21.11514 PMC23523

[56] Lee S, Lee S, Kim JH, Kim JH, Seo JW, et al (2011) Lipocalin-2 Is a chemokine inducer in the central nervous system. Role of chemokine ligand 10 (CXCL10) in lipocalin-2-induced cell migration. J Biol Chem 286: 43855–43870. 22030398 10.1074/jbc.M111.299248 PMC3243551

[57] Lee W-K, Thevenod F (2006) A role for mitochondrial aquaporins in cellular life-and-death decisions? Am J Physiol Cell Physiol 291: C195– C202. 16624989 10.1152/ajpcell.00641.2005

[58] Jeffrey KL, Brummer T, Rolph MS, Liu SM, Callejas NA, et al (2006) Positive regulation of immune cell function and inflammatory responses by phosphatase PAC-1. Nat Immunol 7: 274–283. 16474395 10.1038/ni1310

[59] Pietr M, Kozela E, Levy R, Rimmerman N, Lin YH, et al (2009) Differential changes in GPR55 during microglial activation. FEBS Letters 583: 2071–2076. 19464294 10.1016/j.febslet.2009.05.028

[60] McHugh D, Hu SS, Rimmerman N, Juknat A, Vogel Z, et al (2010) *N*-arachidonoyl glycine, an abundant endogenous lipid, potently drives directed cellular migration through GPR18, the putative abnormal cannabidiol receptor. BMC Neurosci 11: 44. 20346144 10.1186/1471-2202-11-44 PMC2865488

[61] Rimmerman N, Bradshaw HB, Kozela E, Levy R, Juknat A, et al (2012) Compartmentalization of endocannabinoids into lipid rafts devoid of caveolin-1 in a microglial cell line. Brit J Pharmacol 165: 2436–2449. 21449981 10.1111/j.1476-5381.2011.01380.x PMC3423248

[62] Kiss-Toth E, Bagstaff SM, Sung HY, Jozsa V, Dempsey C, et al (2004) Human tribbles, a protein family controlling mitogen-activated protein kinase cascades. J Biol Chem 279: 42703–42708. 15299019 10.1074/jbc.M407732200

[63] Ohoka N, Yoshii S, Hattori T, Onozaki K, Hayashi H (2005) TRB3, a novel ER stress-inducible gene, is induced via ATF4-CHOP pathway and is involved in cell death. EMBO J 24: 1243–1255. 15775988 10.1038/sj.emboj.7600596 PMC556400

[64] Du K, Herzig S, Kulkarni RN, Montminy M (2003) TRB3: a tribbles homolog that inhibits Akt/PKB activation by insulin in liver. Science 300: 1574–1577. 12791994 10.1126/science.1079817

[65] Wu M, Xu LG, Zhai Z, Shu HB (2003) SINK is a p65-interacting negative regulator of NF-kappaB-dependent transcription. J Biol Chem 278: 27072–27079. 12736262 10.1074/jbc.M209814200

[66] Sizemore N, Leung S, Stark GR (1999) Activation of phosphatidylinositol 3-kinase in response to interleukin-1 leads to phosphorylation and activation of the NF-kappaB p65/RelA subunit. Mol Cell Biol 19: 4798–4805. 10373529 10.1128/mcb.19.7.4798 PMC84278

[67] Dos Santos S, Delattre AI, De Longueville F, Bult H, Raes M (2007) Gene expression profiling of LPS-stimulated murine macrophages and role of the NF-kappaB and PI3K/mTOR signaling pathways. Ann N Y Acad Sci 1096: 70–77. 17405917 10.1196/annals.1397.071

[68] Kim J, Cha Y-N, Surh Y-J (2010) A protective role of nuclear factor-erythroid 2-related factor-2 (Nrf2) in inflammatory disorders. Mutat Res 690: 12–23. 19799917 10.1016/j.mrfmmm.2009.09.007

[69] Takahashi Y, Ohoka N, Hayashi H, Sato R (2008) Trb3 suppresses adipocyte differentiation by negatively regulating PPARγ transcriptional activity. J Lipid Res 49: 880–892. 18187772 10.1194/jlr.M700545-JLR200

[70] O′Sullivan SE, Sun Y, Bennett AJ, Randall MD, Kendall DA (2009) Time-dependent vascular actions of cannabidiol in the rat aorta. Eur J Pharmacol 612: 61–68. 19285060 10.1016/j.ejphar.2009.03.010

[71] Necela BM, Su W, Thompson EA (2008) Toll-like receptor 4 mediates cross-talk between peroxisome proliferator-activated receptor γ and nuclear factor-κB in macrophages. Immunology 125: 344–358. 18422969 10.1111/j.1365-2567.2008.02849.x PMC2669138

[72] Nguyen T, Sherratt PJ, Nioi P, Yang CS, Pickett CB (2005) Nrf2 controls constitutive and inducible expression of ARE-driven genes through a dynamic pathway involving nucleocytoplasmic shuttling by Keap1. J Biol Chem 280: 32485–32492. 16000310 10.1074/jbc.M503074200

[73] Talalay P, Dinkova-Kostova AT, Holtzclaw WD (2003) Importance of phase 2 gene regulation in protection against electrophile and reactive oxygen toxicity and carcinogenesis. Adv Enzyme Regul 43: 121–134. 12791387 10.1016/s0065-2571(02)00038-9

[74] Galon J, Franchimont D, Hiroi N, Frey G, Boettner A, et al (2002) Gene profiling reveals unknown enhancing and suppressive actions of glucocorticoids on immune cells. Faseb J 16: 61–71. 11772937 10.1096/fj.01-0245com

[75] Liby KT, Yore MM, Sporn MB (2007) Triterpenoids and rexinoids as multifunctional agents for the prevention and treatment of cancer. Nat Rev Cancer 7: 357–369. 17446857 10.1038/nrc2129

[76] Tanaka T, De Luca LM (2009) Therapeutic potential of rexinoids in cancer prevention and treatment. Cancer Res 69: 4945–4947. 19509234 10.1158/0008-5472.CAN-08-4407

