# Supplementary figures and images for: Microarray and Pathway Analysis Reveal Distinct Mechanisms Underlying Cannabinoid-Mediated Modulation of LPS-Induced Activation of BV-2 Microglial Cells
Source: PLoS One. 2013 Apr 24;8(4):e61462. doi: 10.1371/journal.pone.0061462 (PMC3634783; doi:10.1371/journal.pone.0061462)

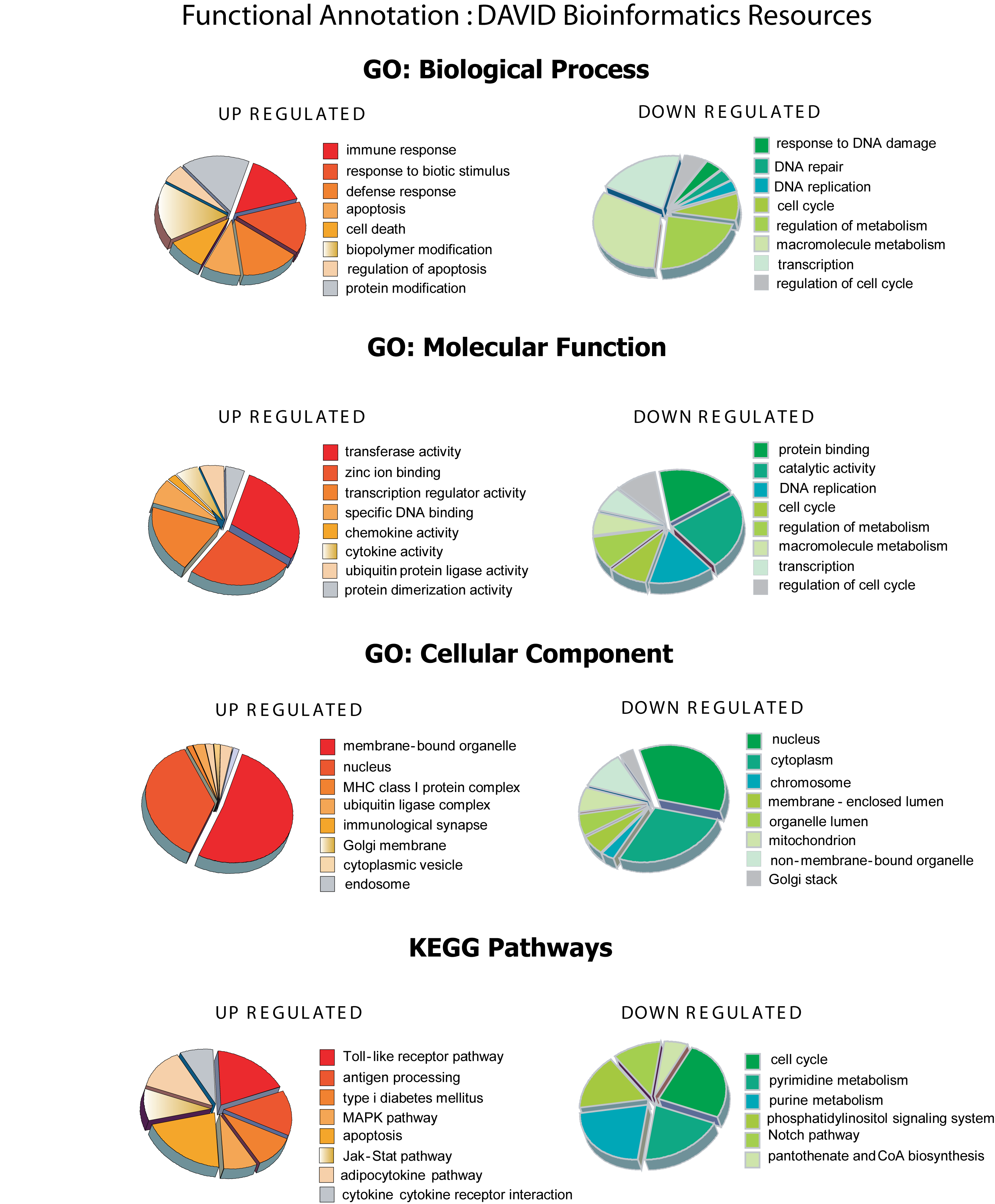

Supplement: Figure S1 — Functional and pathway annotations according to DAVID Bioinformatics Resources of affected gene products following LPS treatment. Gene Ontology (GO) analysis was performed separately for upregulated (red) and downregulated (green) genes, to identify functional categories and cluster of genes significantly affected by LPS treatment. The GO terms were arbitrarily chosen at various levels within the ontology to avoid redundant allocation. Categories shown are significantly represented at p≤0.05. The bottom pie charts represent the cellular pathways according to KEGG database. (TIF) [file pone.0061462.s001.tif]

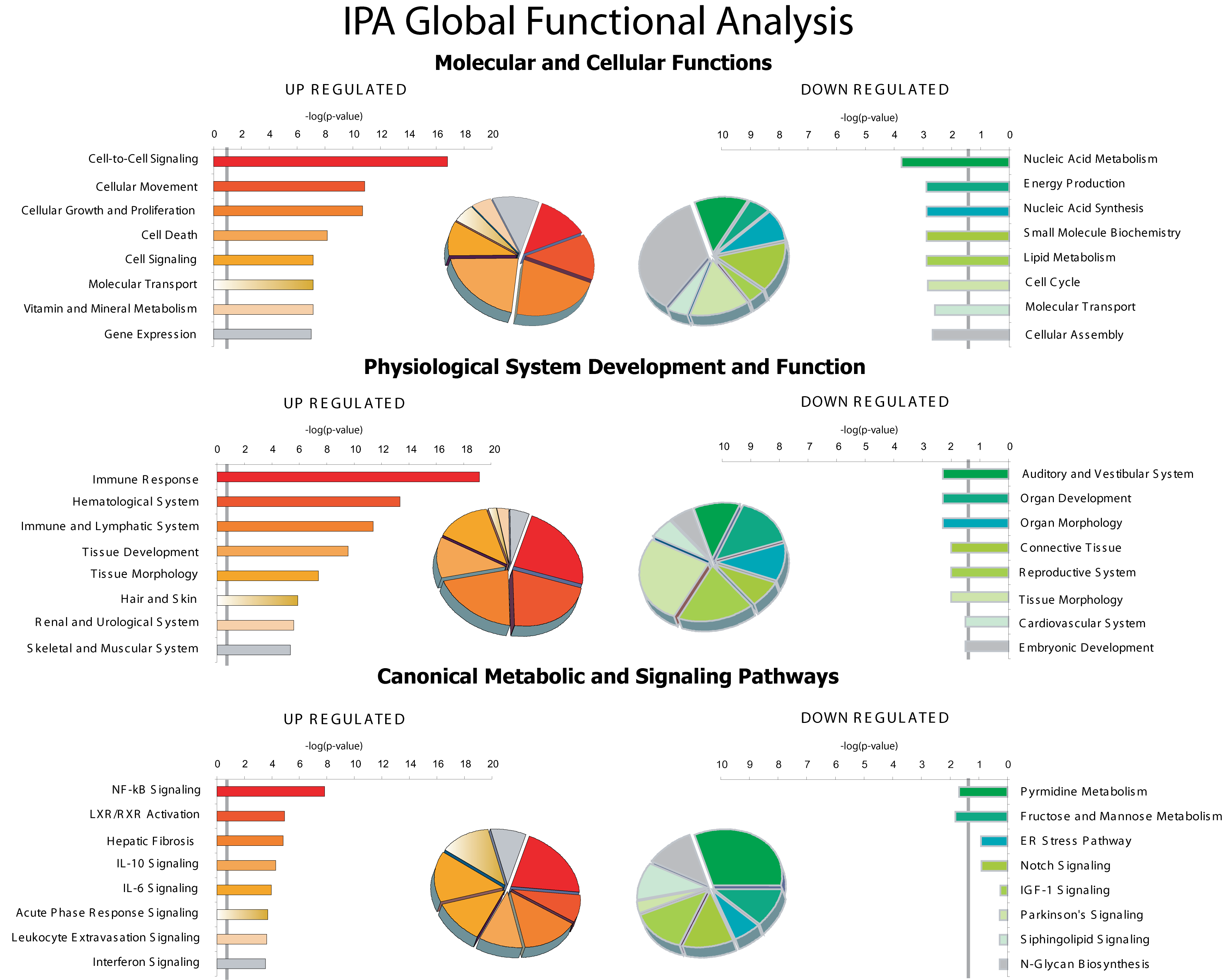

Supplement: Figure S2 — Distribution of differentially expressed genes by global functional annotation and pathway association according to IPA analysis. IPA global functional and pathway analysis was used to examine the enriched functional classes of upregulated (warm colors) and downregulated (cold colors) gene transcripts among the LPS-stimulated genes. The y axis shows the top 8 most representative high-level functions and canonical pathways associated with genes regulated in LPS-treated BV-2 cells. The x axis displays the mean p-value for each associated high-level function and canonical pathway in a -log scale. Increasing value of −log (significance) indicates increased confidence for each category. The vertical gray line in each plot indicates p = 0.05. (TIF) [file pone.0061462.s002.tif]
