# Supplementary material for: Microarray and Pathway Analysis Reveal Distinct Mechanisms Underlying Cannabinoid-Mediated Modulation of LPS-Induced Activation of BV-2 Microglial Cells
Source: PLoS One. 2013 Apr 24;8(4):e61462. doi: 10.1371/journal.pone.0061462 (PMC3634783; doi:10.1371/journal.pone.0061462)
Supplement: Table S1 — Primer sequences for qPCR. a Amplicon length in base pairs; b Genbank accession number of cDNA and corresponding gene, available at http://www.ncbi.nlm.nih.gov/http://www.ncbi.nlm.nih.gov/; c FW, forward primer; RV, reverse primer (DOC) [file pone.0061462.s003.doc]

**Table S1.** Primer sequences used for qPCR

| **Genes** | | **Sequence (5′ → 3′)** | **Length (bp)****^a^** | **Accession****[b](http://www.sciencedirect.com/science?_ob=ArticleURL&_udi=B6WDF-4H0BSYS-1&_user=48161&_coverDate=09%2F21%2F2005&_rdoc=1&_fmt=full&_orig=search&_cdi=6765&_sort=d&_docanchor=&view=c&_acct=C000005078&_version=1&_urlVersion=0&_userid=48161&md5=b53f77d6e3c3d7dcb106335c6673aa48#tblfn2)** |
| --- | --- | --- | --- | --- |
| ***B2mg*** | FW**^c^**  RV | ATG GGA AGC CGA ACA TAC TG  CAG TCT CAG TGG GGG TGA AT | 176 | [NM_009735.3](http://www.ncbi.nlm.nih.gov/nuccore/NM_009735.3) |
| ***Ccl2*** | FW  RV | ACT GCA TCT GCC CTA AGG TCT TCA  TTC ACT GTC ACA CTG GTC ACT CCT | 131 | [NM_011333.3](http://www.ncbi.nlm.nih.gov/nuccore/NM_011333.3) |
| ***Ccl12*** | FW  RV | ATC AGT CCT CAG GTA TTG GCT GGA  TGG CTG CTT GTG ATT CTC CTG TAG | 125 | [NM_011331.2](http://www.ncbi.nlm.nih.gov/nuccore/NM_011331.2) |
| ***Cxcl10*** | FW  RV | TTA ACT GGA GTG AAG CCA CGC ACA  TCA CCT TTC AGA AGA CCA AGG GCA | 128 | [NM_021274.2](http://www.ncbi.nlm.nih.gov/nuccore/NM_021274.2) |
| ***Aqp9*** | FW  RV | TGA AGG GAC AAG GTA GCC GTT TGA  AAA CAG TTG GCA GTG AAG GCA CAC | 193 | [NM_022026.2](http://www.ncbi.nlm.nih.gov/nuccore/NM_022026.2) |
| ***Mcoln2/Trpml2*** | FW  RV | AGC AGT CGG CTC AAA GAT TGT CCT  ACA AAC GAC CAG ACT CCT GGT GAA | 175 | [NM_026656.4](http://www.ncbi.nlm.nih.gov/nuccore/NM_026656.4) |
| ***Slc7a11*** | FW  RV | AAA GCA GGT TCC ACA GCG AAG T  TGG CCA GCT CCG CAA ATG AAA T | 227 | [NM_011990.2](http://www.ncbi.nlm.nih.gov/nuccore/NM_011990.2) |
| ***Cdkn1a*** | FW  RV | TGC CTG GTT CCT TGC CAC TTC TTA  TTC ACT GTC ATC CTA GCT GGC CTT | 122 | [NM_007669.4](http://www.ncbi.nlm.nih.gov/nuccore/NM_007669.4) |
| ***Dusp1*** | FW  RV | ATT TGC TGA ACT CGG CAC ATT CGG  GGT GGG TGT GTC AAG CAT GAA GTT | 154 | [NM_013642.3](http://www.ncbi.nlm.nih.gov/nuccore/NM_013642.3) |
| ***Dusp2*** | FW  RV | AGA TGG TGG AGA TAA GTG CCT GGT  AAT CAG GTA TGC CAG GCA GAT GGT | 137 | [NM_010090.2](http://www.ncbi.nlm.nih.gov/nuccore/NM_010090.2) |
| ***Sqstm1/p62*** | FW  RV | ACC CTC CAC CAT TGT GAT AGT GCT  AAT GCC AAG ACA CTG GGC CTA TCT | 109 | [NM_011018.2](http://www.ncbi.nlm.nih.gov/nuccore/NM_011018.2) |
| ***Ebi2/Gpr183*** | FW  RV | ATG CTG GTC ATG ACA GAG GTG ACA  AGG CAC TAC CAG CTG AAC AGA AGT | 198 | [NM_183031.2](http://www.ncbi.nlm.nih.gov/nuccore/NM_183031.2) |
| ***Ptgir*** | FW  RV | AGT GCT CCT GCT GAT GTT TCT CCT  AAA GTT CTG CTG TGT GGT CCA AGC | 165 | [NM_008967.3](http://www.ncbi.nlm.nih.gov/nuccore/NM_008967.3) |
| ***Gpr55*** | FW  RV | ACA GCT GAA AGC CTC ACT CTC CAT  AAA GCA CAC GAA GTC TCC TGG GAT | 132 | [NM_001033290.2](http://www.ncbi.nlm.nih.gov/nuccore/NM_001033290.2) |
| ***Cnr2*** | FW  RV | AGG AGG AAG TGC TTG GTT CTG TCA  CCA AAG CTG GTG CAG GAA TTC ACA | 107 | [NM_009924.3](http://www.ncbi.nlm.nih.gov/nuccore/NM_009924.3) |
| ***Tlr2*** | FW  RV | ATT CCC ATT GGG TGG AGA ACC TCA  CAA TGA TCC ATT TGC CCG GAA CGA | 108 | [NM_011905.3](http://www.ncbi.nlm.nih.gov/nuccore/NM_011905.3) |
| ***Trib3*** | FW  RV | AAG ACT TGG CTG TGG GAT TCA AGC  AGA ACA GGG CCT GAG ATT GTC TGT | 190 | [NM_175093.2](http://www.ncbi.nlm.nih.gov/nuccore/NM_175093.2) |
| ***Hmox1*** | FW  RV | GTG GCC TGA ACT TTG AAA CCA GCA  ACA GCA GTC GTG GTC AGT CAA CAT | 130 | [NM_010442.2](http://www.ncbi.nlm.nih.gov/nuccore/NM_010442.2) |
| ***Pparg1*** | FW  RV | ATC CGT AGA AGC CGT GCA AGA GAT  TGA ATC CTT GGC CCT CTG AGA TGA | 179 | [NM_001127330.1](http://www.ncbi.nlm.nih.gov/nuccore/NM_001127330.1) |
| ***Pparg2*** | FW  RV | AGG GCG ATC TTG ACA GGA AAG ACA  TAC TCT GTG ATC TCT TGC ACG GCT | 197 | [NM_011146.3](http://www.ncbi.nlm.nih.gov/nuccore/NM_011146.3) |

[Full-size table](http://www.sciencedirect.com/science?_ob=MiamiCaptionURL&_method=retrieve&_udi=B6WDF-4H0BSYS-1&_image=tbl1&_ba=&_user=46001&_rdoc=1&_fmt=full&_orig=search&_cdi=6765&view=c&_isTablePopup=Y&_acct=C000005078&_version=1&_urlVersion=0&_userid=48161&md5=017caea86eb2ba6c08db725eabbfe240)

^a^ Amplicon length in base pairs; ^b^ Genbank accession number of cDNA and corresponding gene,

available at [http://www.ncbi.nlm.nih.gov/http://www.ncbi.nlm.nih.gov/](http://www.sciencedirect.com/science?_ob=RedirectURL&_method=externObjLink&_locator=url&_cdi=6765&_plusSign=%2B&_targetURL=http%253A%252F%252Fwww.ncbi.nlm.nih.gov%252F) ; ^c^ FW, forward primer;

RV, reverse primer
